# (1-(4-(5-Phenyl-1,3,4-oxadiazol-2-yl)phenyl)-1*H*-1,2,3-triazol-4-yl)-methylenyls α,ω-Bisfunctionalized 3- and 4-PEG: Synthesis and Photophysical Studies

**DOI:** 10.3390/molecules28135256

**Published:** 2023-07-06

**Authors:** Mohammed S. Mohammed, Igor S. Kovalev, Natalya V. Slovesnova, Leila K. Sadieva, Vadim A. Platonov, Grigory A. Kim, Rammohan Aluru, Alexander S. Novikov, Olga S. Taniya, Valery N. Charushin

**Affiliations:** 1 Chemical Engineering Institute, Ural Federal University, 19 Mira St., 620002 Yekaterinburg, Russia; mmokhammed@urfu.ru (M.S.M.); i.s.kovalev@urfu.ru (I.S.K.); n.v.slovesnova@urfu.ru (N.V.S.); vadim.platonov@urfu.ru (V.A.P.); raluru@urfu.ru (R.A.); olga.tania@urfu.ru (O.S.T.); v.n.charushin@urfu.ru (V.N.C.); 2 Department of Pharmacy and Chemistry, Ural Medical University, 3 Repina St., 620028 Yekaterinburg, Russia; 3 I. Ya. Postovsky Institute of Organic Synthesis of RAS (Ural Division), 22/20 S. Kovalevskoy/Akademicheskaya St., 620137 Yekaterinburg, Russia; kim-g@ios.uran.ru; 4 Institute of Natural Sciences and Mathematics, Ural Federal University, 19 Mira St., 620002 Yekaterinburg, Russia; 5 Institute of Chemistry, Saint Petersburg State University, 7/9 Universitetskaya Nab., 199034 Saint Petersburg, Russia; a.s.novikov@spbu.ru; 6 Research Institute of Chemistry, Peoples’ Friendship University of Russia (RUDN University), 6 Miklukho-Maklaya Street, 117198 Moscow, Russia

**Keywords:** oxadiazole, PEGs, bola molecules, “click” reactions, fluorescence, sensor, nitro-explosive components, pentaerythritol tetranitrate, Hg^2+^, fluorescence quenching

## Abstract

Two new azaheterocycle-based bolas, such as (1-(4-(5-phenyl-1,3,4-oxadiazol-2-yl)phenyl)-1*H*-1,2,3-triazol-4-yl)-methylenyls α,ω-bisfunctionalized PEGs, were prepared via Cu-catalyzed click reaction between 2-(4-azidophenyl)-5-(aryl)-oxadiazole-1,3,4 and terminal ethynyls derived from PEG-3 and PEG-4. Due to the presence of two heteroaromatic cores and a PEG linker, these bola molecules are considered as promising fluorescent chemosensors for electron-deficient species. As a result of a well-pronounced “turn-off” fluorescence response towards common nitro-explosive components, such as 2,4-dinitrotoluene (DNT) and 2,4,6-trinitrotoluene (TNT), hard-to-detect pentaerythritol tetranitrate (PETN), as well as Hg^2+^ cation was observed.

## 1. Introduction

One of the most important tasks of modern synthetic organic chemistry is to obtain new compounds that will find wide application in various industries and medicine. These could be fluorophores that contain cyclic azole and azine fragments as pharmacophores. The targeted synthetic design of functional bola-type scaffolds and the study of their response to conformational changes or the presence of (bio)analytes are of key importance for their application in a wide variety of fields. Typical bola scaffolds are composed of two functional units acting as receptors and/or fluorophores and connected by alkyl or heteroatom-containing chains [1]. Bola-type scaffolds have attracted a great deal of attention for their application in biology and pharmacy, including in drug and gene transport systems [2,3,4,5], chemical sensing, biomedical applications, and molecular recognition [6,7,8]. Chemosensor-assisted fluorescence detection is one of the most convenient techniques used for the recognition of various (bio)analytes, including explosives. Noticeable advantages of fluorescence methods are their simplicity, fast detection time, and the possibility of the fine structural tuning of chemosensor/sensory material to improve selectivity and/or desired photophysical properties. In comparison with other physical methods, such as chromatographic methods [9], HPLC-electron capture detection (HPLC-ECD) [10], mass spectrometry [11,12], photoacoustic spectroscopy [13], and electrochemical methods [14,15], fluorescence-chemosensor-based methods are inexpensive, mobile, and accurate, which are crucial factors for their application in the development of highly affordable and reliable sensing devices. Among various types of bola-type scaffolds, aryl-substituted triazole- and oxadiazole-based scaffolds were selected by us due to a bright emission in the visible region [16,17,18]. 

Compared to other commonly used nitroaromatic explosives, the detection of aliphatic explosives, such as pentaerythritol tetranitrate (PETN)—one of the major components in plastic explosives—is a challenging task due to the low vapor pressure of PETN (7,17 ppt) and its less pronounced electron-deficient character (µ = 0.0033D) [19]. Another problem for the fluorescent detection of PETN is its high-lying LUMO energy level, and to ensure efficient “chemosensor-analyte” PET transfer, high-LUMO energy level chemosensors are required [20].

We recently reported the synthesis of several PAH-containing bola-type chemosensors for the fluorescent “turn-off” detection of explosives in aqueous solutions. In this manuscript, we wish to report our results on the synthesis of bola-type 1,2,3-triazole-based fluorophores/chemosensors and on the study of their photophysical properties and fluorescence response towards some nitro-explosives and metal cations.

## 2. Results

### 2.1. Synthesis

**Scheme 1:**
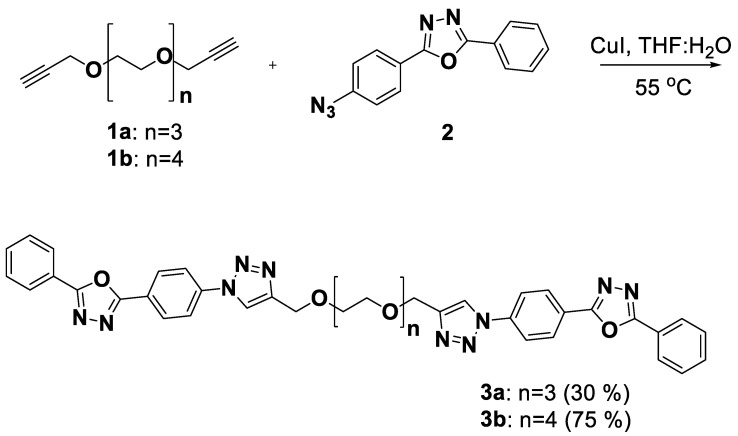
Synthesis of α,ω-bisfunctionalized by the (1-(4-(5-phenyl-1,3,4-oxadiazol-2-yl)phenyl)-1*H*-1,2,3-triazol-4-yl)-methylenyls PEGs **3a-b**.

The synthetic strategy for the preparation of target compounds **3a-b** is presented below (Scheme 1). This strategy involves a copper-catalyzed azide-alkyne cycloaddition. Thus, an azide component, 4-azidophenyloxadiazole **2**, was prepared by means of modified Sandmeyer reaction [21,22]. Additionally, the ethynyl components, such as alkyne-terminated ethylenglycols **1a-b,** were prepared as reported previously [23] (Scheme 1). As a final step, Cu(I)-promoted azido-alkyne coupling (CuAAC) was carried out by means of the heating of compounds **1** and **2** in THF-H_2_O solution in the presence of CuI to afford the target products **3a-b** in 30–75% yields. The structures of all the obtained compounds were confirmed by means of ^1^H and ^13^C NMR spectroscopy, mass spectrometry, and elemental analysis (see ESI for details).

### 2.2. Photophysical Studies

**Figure 1:**
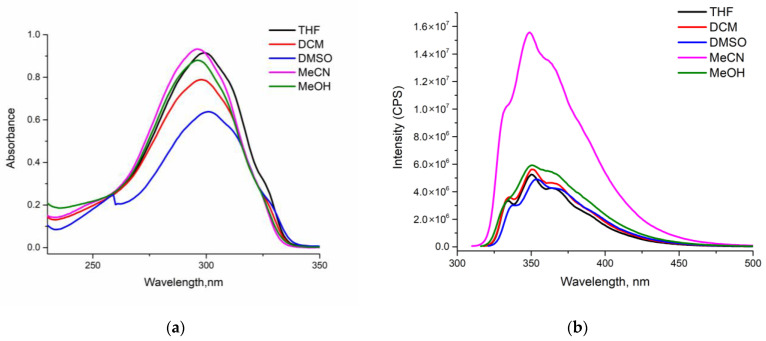
Absorption (**a**) and emission (**b**) spectra of the probe **3a** (10^−5^ M) in different solvents.

**Figure 2:**
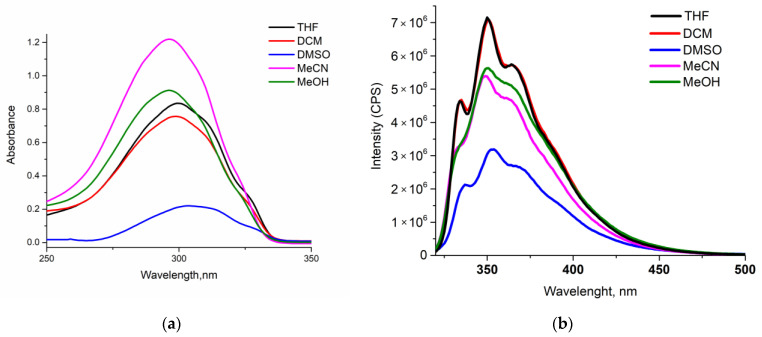
Absorption (**a**) and emission (**b**) spectra of the probe **3b** (10^−5^ M) in different solvents.

Next, the photophysical studies of the **3** compounds were carried out. At concentrations below 2 × 10^−5^ M, both samples, **3a-b,** were well soluble both in medium and high polar aprotic solvents (THF, dichloromethane, DMSO, acetonitrile) and in high-polar protic solvents such as methanol. In absorption spectra, intensive absorption maxima were observed around 300 nm. Upon excitation at 300 nm, an intensive fluorescence in a range from 325 to 425 nm was observed in emission spectra. The absorption and emission spectra of the samples are presented in Figure 1 and Figure 2, respectively.

Both compounds **3a-b** can be considered as structural analogs of well-reported POPOP dye. The absorption and emission properties of both fluorophores and the POPOP dye (as a reference sample) in a solution of THF are presented in Table 1. Thus, both fluorophores exhibited 2 similar absorption bands, the high-energy band at λ_max_ 216 nm and the lower energy band at λ_max_ 300 nm, which correspond to the S_0_→S_2_ and S_0_→S_1_ transitions. Both samples had a dominative transition band of S_0_ → S_1_ with _ε__M_ > 80,000 M^−1^ sm^−1^. These high molecular absorption extinction coefficients (_ε__M_ = 30,800–91,500 M^–1^ sm^–1^), as well as the high values of the oscillator strength (f = 1.47–1.62, Table S6), correspond well with strong π-π*-transitions, with little change in geometry, between electronic ground and excited states.

As shown in Figure 1a and Figure 2a, the polarity of the solvent did not significantly influence the position of absorption peaks, indicating the weakly polar nature of POPOP-like **3** samples in a ground state. On the other hand, fluorescence spectra were more sensitive to solvent polarity. Thus, the increase in the polarity of the solvent resulted in the slight bathochromic shift of the emission maxima along with a significant increase in emission intensity (Figure 1b and Figure 2b). At the same time, structured emission spectra of the samples **3a-b**, with high PLQY > 60% and Stokes shifts < 100 nm (Δν = 50 ÷ 58 nm), led to our conclusion about the predominance of the state of fluorophores in local excitation (LE), which correlates well with the data of DFT calculations (see Section 2.3) [24]. As shown in Table 1, samples **3a-b** had high molar extinction coefficients in the long wavelength region (ε_M_ > 80,000 M^−1^ cm^−1^) and fluorescence quantum yields above 60%, suggesting that **3a,b** are good candidates for practical application as fluorescent sensors/probes.

**Table 1:** Photophysical properties of the probes **3a-b** (10^−5^ M) and POPOP in THF in solution.

Probes	λ_abs_^max^, nm (ε_M_, 10^5^ M^−1^ cm^−1^) ^1^	λ_em_^max^, nm ^2^	Stokes Shift, nm	τ_av_, ns ^3^	*Φ_f_*, % ^4^
**3a**	216 (0.308) 299 (0.915)	335, 350, 364	51	1.08	61.7
**3b**	216 (0.360) 300 (0.835)	335, 350, 364	50	1.29	86.6
**POPOP ^5^**	348, 361, 379	393, 416, 438	-	-	96.0

Notes: ^1^ absorption spectra were measured at r.t. in THF in range from 230 to 350 nm; ^2^ emission spectra were measured at r.t. in THF; ^3^ weighted average decay time τ_av_ = Σ (τ_i_ × α_i_) in THF (LED 310 nm); ^4^ absolute quantum yields were measured using the integrating sphere of a HORIBA FluoroMax-4 at r.t. in THF; ^5^ reference [25].

Next, time-resolved fluorescence studies were carried out. Thus, for both samples, all the time-resolved fluorescence decays could be fitted with double exponential components whose amplitudes αi and time constants τ_i_, are presented in Table S1. We assume that for the samples **3a-b,** the fast component may be related to the processes of rapid fluorescence quenching that does not reach the stabilized LE geometry.

To study the fluorescence “turn-off” response of the **3** fluorophores to nitro-containing analytes in various media, the most effective photoinduced electronic transition, LE, was selected [26]. To study the fluorescence response, the most common nitroaromatic explosive components, DNT and TNT, were selected, as well as low volatile and hard-to-to detect nitro-explosives such as pentaerythritol tetranitrate (PETN). Fluorescence studies were carried out in MeCN solution at 296 K by using concentrations of the chemosensors **3** of 10^–6^ M in order to avoid self-quenching. To calculate the quenching efficiency of the chemosensors **3a-b,** the Stern–Volmer mathematical model was used:

I_0_/I = 1 + K_sv_ × [Q] (1)

where I_0_ and I are fluorescence intensity before and after the addition of a nitroaromatic compound (quencher); Q is the concentration of the nitroaromatic compound, mol/L; and K_sv_ is the value of the constant, M^−1^.

Based on the results of fluorescence quenching experiments, the Stern–Volmer quenching constants (K_sv_) of the “**3a**/**3b** sample-nitro-analyte” were estimated by means of measuring the slope of the SV plot, and the constants’ value was found to be as high as ~1.0 × 10^−4^ M^−1^ for the nitroaromatic aromatic quenchers and 0.5 × 10^−4^ M^−1^ for the PETN (Table 2). The minimum limit of detection (LOD) was calculated based on the fluorometric titration data for **3a-b** [27]. The high order of binding constants (~0.7 × 10^4^ M^−1^), with detection limits of 3 × 10^−7^ M (~95 ppb), identified the sample **3b** as the most promising for the qualitative and quantitative detection of nitro compounds of various natures. The obtained data are comparable with values of quenching constants and LOD for the previously reported chromophores/chemosensors [28]. 

To confirm the selectivity of **3a,b** towards nitroaromatic compounds, their fluorescent response to a nonexplosive compound (such as highly electron-deficient benzoquinone (BQ)) was studied and, as a result, no fluorescence quenching was observed. 

The linear quenching response on the SV plots for the **3** chemosensors (Figures S3–S26) suggests that only one quenching mechanism prevails. The static quenching assumes the formation of stable nonfluorescent complexes’ “chemosensor-quencher”. However, we did not observe the formation of such a complex between the chemosensor **3b** and TNT during the UV–Vis spectrophotometric titration experiments (Figure S27). The dynamic quenching model was also ruled out as, during the time-resolved fluorescence quenching, no decrease of the lifetime of the chemosensor **3b** upon increasing the concentration of TNT was observed (Table S3, Figures S28 and S29). Based on these observations, a false static quenching model via photoinduced electron transfer (PET) was suggested [29]. In this case, unlike for the static quenching model [30], no stable nonfluorescence adduct formed, but fluorescence quenching takes place due to the presence of a quencher on a periphery (action volume) of a chemosensor/fluorophore [31].

### 2.3. DFT Calculations

To support the experimental results, DFT calculations were carried out at the CAM-B3LYP/6-31G*//PM6-level of theory by using the Gaussian-09 [32] program package. No symmetry restrictions were applied during the geometry optimization procedure. The solvent effects were taken into account using the SMD (solvation model based on density) continuum solvation model suggested by Truhlar and coworkers [33]. The Hessian matrices were calculated for all optimized model structures to prove the location of correct minima on the potential energy surface (no imaginary frequencies were found in all cases). The Cartesian atomic coordinates for all optimized equilibrium model structures are presented in the attached xyz-files. Hole–electron analysis was carried out in Multiwfn program, Version 3.7 [34].

**Figure 3:**
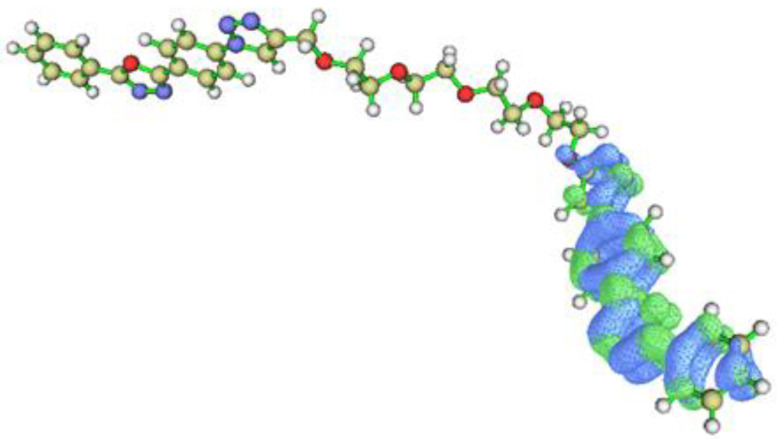
Electron density difference patterns between S_0_ and S_1_ state for **3b**.

As a first step, in order to evaluate the internal characteristics of the processes of electronic excitation, an analysis of the difference in electron density was carried out (Figure 3). According to Table S6, all the main absorption peaks of **3a-b** belong to the S_0_-S_1_ transition; therefore, the analysis of the electron density difference is built between the S_0_ and S_1_ states and refers to the π–π* transition.

As shown in Figure 3, blue and green isosurfaces (isovalue = 0.001) represent hole and electron distributions, respectively. The highly overlapped isosurfaces and the fact that the hole and electron occupy a similar spatial region prove that the S_0_–S_1_ transition is a local excitation process, which agrees well with the experimental data. For both samples (**3a,b)**, the electron density isosurfaces are concentrated within one aromatic part of the molecule, which is associated with the violation of the initial conjugation due to the presence of the PEG bridge. Thus, special optical properties of sensors are associated with different distributions of electronic excitation. 

In order to study the sensing mechanism, optimized configurations of adducts of interaction of samples with analytes, which are difficult to obtain from experiments, were considered in detail (Figure S33). When optimizing the adduct for the sample **3a**, the nitroanalyte molecules turned out to be oriented with respect to the polyethylene group. For the sample **3b**, NAC were oriented to the 1,2,3-triazole and phenylene fragments, and PETN was oriented to the polyethylene group and the phenylene fragment.

**Figure 4:**
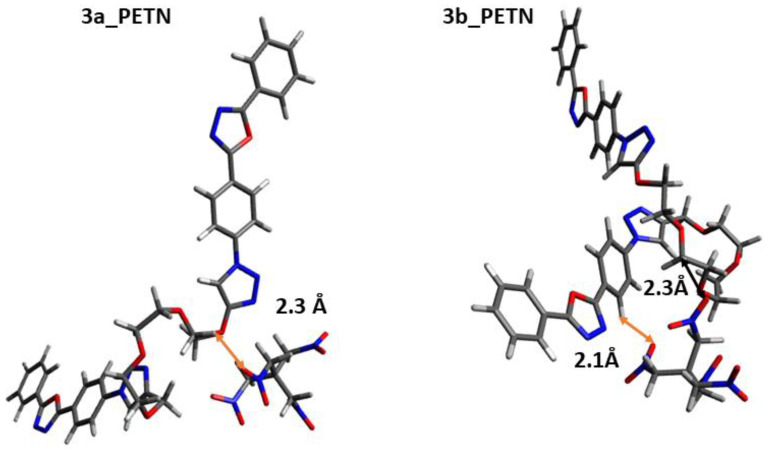
Optimized configurations of **3a-PETN** and **3b-PETN** adducts.

Figure 4 shows that in the optimized state, the closest distance between the hydrogen of the phenyl fragment of the sample **3b** and the oxygen atoms of the PETN nitro groups was only 2.1 Å, and the distance between the hydrogen atoms of the methylene group and the oxygen atoms of the PETN nitro groups was 2.3 Å, which indicates a strong interaction between the sensor and the analyte. For comparison, the closest distance between the hydrogen atoms of the methylene group **3a** and the oxygen atoms of the nitro group of PETN was only 2.3 Å (Figure 4, Table 3).

Gibbs electronic energies were calculated to evaluate the optimized model structures (Table 4). Interactions of samples with explosives were considered as hypothetical supramolecular association processes. The results of DFT calculations showed that the “**3b**-TNT” and “**3b**-PETN” adducts were the most stable, with a lowest energy of −3672.38 and −3541.75 a.u., respectively. This demonstrates the most energetically favorable combination between the sample **3b** with aromatic (TNT) and nitroaliphatic (PETN) analytes, which correlates well with both the K_SV_ values and DFT-optimized adduct configurations.

**Figure 5:**
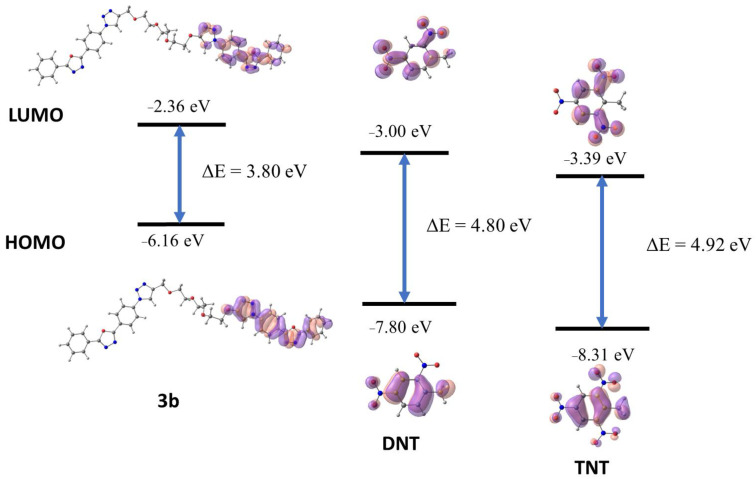
Molecular orbitals and energy levels of **3b**, DNT, and TNT calculated at the B3LYP_631pGs-level of theory.

**Figure 6:**
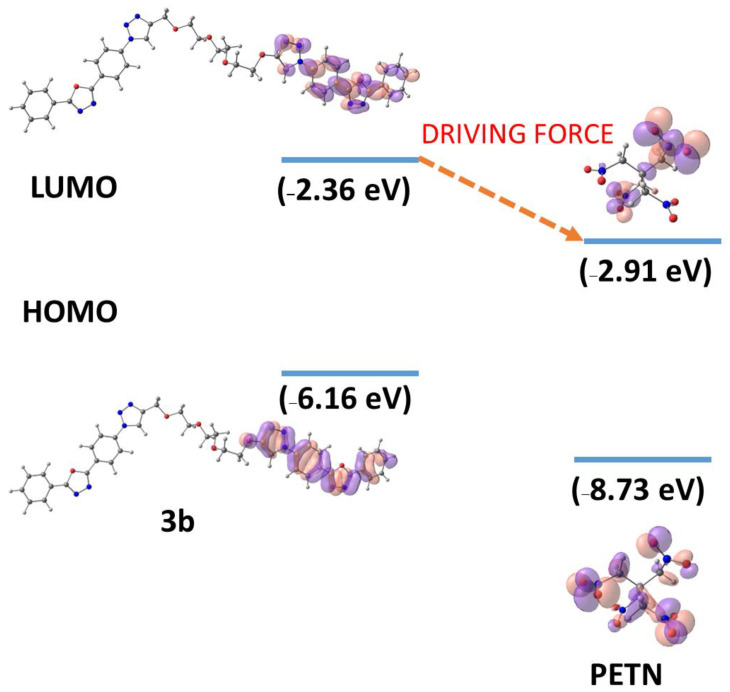
DFT Calculations for the HOMO and LUMO energies for the sensor 3b and PETN.

The process of photoinduced electron transfer (PET) occurs from the LUMO of the excited sample to the LUMO of NAC, followed by the reverse nonradiative electron transfer to the HOMO of the sample, which leads to fluorescence quenching [28]. Therefore, higher fluorescence quenching efficiency of samples **3a-b** was observed for DNT/TNT nitroaromatic compounds with a marginal electron deficit due to their relatively lower LUMO energy levels (LUMO (DNT) 3.00 eV, LUMO (TNT) 3.39 eV) (Figure 5). Because there was no overlap between the emission spectra of chemosensors **3a-b** and the absorption spectrum of the PETN, no energy transfer between them had occurred [35]. Therefore, due to the high LUMO energy level of chemosensors **3a-b** (2.35 and 2.36 eV, respectively), a more pronounced driving force of the PET process from the LUMO level of the chemosensor **3b** to the LUMO level of PETN takes place (Table 5, Figure 6).

### 2.4. Recognition of Hg^2+^ via Fluorescence “Turn Off” Process

Visual detection of highly toxic Hg^2+^ cation is important for food safety and environmental monitoring. Hg^2+^ is known to form stable complexes with azaheterocyclic ligands [36,37,38] and crown ethers [39,40,41]. Therefore, as a final step, we studied the fluorescence response in solution of the chemosensors 3a-b to the Hg^2+^ cation and other metal cations.

Thus, in MeCN:H_2_O [90:10 (vol.%)] solutions of chemosensors 3a-b (2 × 10^−4^ M), upon UV light excitation (λ_max_ = 365 nm), a dramatic fluorescence quenching was observed when an aqueous solution of mercury acetate was added (2 × 10^−2^ M), while for other metal cations, no fluorescence quenching was observed (Figure S34). To obtain more evidence, we carried out fluorescence titration of the solution of sensor 3a (10^−6^ M) by the solution of mercury acetate (2 × 10^−4^ M), and a dramatic fluorescence quenching was observed. Similar to 3a, for the sample 3b, a noticeable fluorescence quenching at a very low concentration of Hg^2+^ was observed. This quenching, calculated by the 3σ limit of detection (LOD) method, was measured as ~2.59 × 10^–6^ M (Table 6, Figure S26). The binding constant for the complex “Hg^2+^:3a” was calculated as a tangent of the slope of the SV plot and amounted to ~1.0 × 10^4^ M^−1^, and the quenching of the fluorescence of 3a by Hg^2+^ was as high as 73%. Therefore, chemosensor 3a can be considered as a highly sensitive fluorescent “turn-off“ probe for Hg^2+^. Selective recognition of cation Hg^2+^ by the sample 3a was also demonstrated by additional experiments in the presence of other metal cations (Figure S34). Thus, after the addition of other metal cations, such as Cu^2+^, Co^2+^, Cd^2+^, Hg^2+^, Sn^2+^, Zn^2+^, Ni^2+^, and Mg^2+^, only in the case of Hg^2+^ was a very clear quenching of the fluorescence of the chemosensor 3a observed. Thus, a very high selectivity of 3a towards Hg^2+^ was confirmed.

Again, static and/or dynamic quenching was suggested. In order to understand the nature of the quenching mechanism, fluorescence lifetime measurements for the sample **3a** in the presence of various concentrations of Hg^2+^ were carried out. As a result, no lifetime decrease upon increasing concentrations of Hg^2+^ was observed, which rules out a dynamic quenching mechanism (Table S4, Figures S31 and S32) and suggests the static quenching mechanism. However, upon UV–Vis spectrophotometric titration studies, only subtle changes in the UV spectra of sample **3a** in the presence of various concentrations of Hg^2+^ was observed; thus, no formation of stable molecular complex “3a*Hg^2+^” was confirmed (Figure S30). These results suggest the false static fluorescence quenching mechanism to be predominant for **3a** in the presence of Hg^2+^. Therefore, in the presence of both the nitro-analytes and Hg^2+^ cation the fluorescence quenching of chemosensor **3a** was observed and, in all the cases, the false static mechanism was suggested to be the predominant one.

## 3. Materials and Methods

### Chemical Experiment

Unless otherwise indicated, all common reagents and solvents used were obtained from commercial suppliers without further purification. Melting points were determined on Boetius combined heating stages. TLC and column chromatography were carried out on SiO_2_. Spectra of ^1^H NMR and ^13^C NMR were recorded at room temperature at 400 and 100 MHz, respectively, on a Bruker DRX-400 spectrometer (Bruker BioSpin GmbH, Rheinstetten, Germany) using CDCl_3_ as the solvent. Peaks were labeled as singlet (s), doublet (d), triplet (t), doublet of doublets (dd), doublet of doublets of doublets (ddd), and multiplet (m). The mass spectra (electron impact) were measured on a Shimadzu GCMS-QP2010 Ultra instrument (Shimadzu, Kyoto, Japan). Elemental analyses were performed on a PerkinElmer 2400 Series II CHN Analyzer (PerkinElmer, Waltham, MA, USA). UV–Vis absorption spectra were recorded on the Shimadzu UV-1800 spectrophotometer (Shimadzu, Kyoto, Japan), and emission spectra were measured on a HORIBA FluoroMax-4 (HORIBA Jobin Yvon S.A.S., Longjumeau, France) by using quartz cells with 1 cm path length at room temperature. Absolute quantum yields of luminescence of target compounds in solution were measured by using the Quanta-φ Integrating Sphere of the HORIBA FluoroMax-4 fluorometer (HORIBA Jobin Yvon S.A.S., Longjumeau, France) at room temperature. Fluorometric titration was performed by means of a single-point methodology using a HORIBA FluoroMax-4 fluorometer (HORIBA Jobin Yvon S.A.S., Longjumeau, France). 

General procedure for the synthesis of (1-(4-(5-phenyl-1,3,4-oxadiazol-2-yl)phenyl)-1*H*-1,2,3-triazol-4-yl)-methylenyls α,ω-bisfunctionalized PEG-based fluorophores **3a**-**b**: in a 25 mL Shlenk type flask, 2-(4-azidophenyl)-5-aryl-1,3,4-oxadiazole (2.20 eqv.), copper(I) iodide (0.20 eqv.), and ethynyl component (1 eqv.) were dissolved in 5 mL of THF:H_2_O (4:1) solution. The resulting mixture was heated for 10 h at 65 °C under an argon atmosphere. After the reaction was completed (TLC monitoring), the reaction mixture was diluted with aqueous 10% NH_4_OH (10 mL), and the resulting suspension was filtered. The obtained residue was purified by flash chromatography.

*1,12-Bis(1-(4-(5-phenyl-1,3,4-oxadiazol-2-yl)phenyl)-1H-1,2,3-triazol-4-yl)-2,5,8,11-tetraoxadodecane* **3a.** Yield 103 mg, 30%; ^1^H NMR in CDCl_3_, ppm: 3.71 (s, 4H, 2 × CH_2_O), 3.73–3.76 (m, 4H, 2 × CH_2_O), 3.77–3.81 (m, 4H, 2 × CH_2_O), 4.82 (s, 4H, 2 × CH_2_), 7.54–7.59 (m, 6H, C_6_H_5_), 7.96 (m, 4H, C_6_H_4_), 8.15 (m, 4H, C_6_H_5_), 8.21 (s, 2H, C_2_N_3_H), 8.28 (m, 4H, C_6_H_4_). ^13^C NMR in DMSO-*d*_6_, ppm: 63.2 (1C), 69.0 (1C), 69.6 (1C), 70 (1C), 120.3 (1C), 122 (1C), 123 (1C), 123.1 (1C), 126.6 (1C), 128.1 (1C), 129.2 (1C), 132 (1C), 138.5 (1C), 145.4 (1C), 163 (1C), 164 (1C). EI-MS, *m*/*z* (I, %): 753 (1). Fnd, %: C 63.73, H 4.95, N 18.54; calc. for C_40_H_36_N_10_O_6_, %: C 63.82, H 4.82, N, 18.61.

*1,15-Bis(1-(4-(5-phenyl-1,3,4-oxadiazol-2-yl)phenyl)-1H-1,2,3-triazol-4-yl)-2,5,8,11,14-pentaoxapentadecane* **3b**. Yield 311 mg, 75%; ^1^H NMR in CDCl_3_, ppm: 3.69 (s, 8H, 4 × CH_2_O), 3.70–3.73 (m, 4H, 2 × CH_2_O), 3.75–3.79 (m, 4H, 2 × CH_2_O), 4.80 (s, 4H, 2 × CH_2_), 7.54–7.59 (m, 6H, C_6_H_5_), 7.97 (m, 4H, C_6_H_4_), 8.13- 8.18 (m, 4H, C_6_H_5_), 8.23 (sm, 2H, C_2_N_3_H), 8.29 (sm4H, C_6_H_4_). ^13^C NMR in DMSO-*d*_6_, ppm: 64 (1C), 70 (1C), 70.2 (1C), 70.3 (1C), 121 (1C), 122.6 (1C), 123.6 (1C), 124 (1C), 127.2 (1C), 129 (1C), 130 (1C), 132.5 (1C), 139.3 (1C), 146 (1C), 164 (1C), 165 (1C). EI-MS, *m*/*z* (I, %): [M-C_17_H_12_N_5_O_2_]^+^ = 318 (6). Fnd, %: C 63.24, H 5.10, N 17.69; calc. for C_42_H_40_N_10_O_7_, %: C 63.31, H 5.06, N 17.58.

## 4. Conclusions

The main goal of the research reported herein was to develop new bola-type azole derivatives by using click reaction methodology in order to investigate their photophysical properties and their sensory response to metal cations and nitro-explosives.

In summary, (1-(4-(5-phenyl-1,3,4-oxadiazol-2-yl)phenyl)-1*H*-1,2,3-triazol-4-yl)-methylenyls α,ω-bisfunctionalized polyethylene glycol-based fluorophores **3a-b** were prepared via Cu-catalyzed click reaction. The photophysical properties of these fluorophores as well as the fluorescence “turn-off” response to some common quenchers, such as nitro-analytes and metal cations, were studied. For both sensors, a dramatic fluorescence quenching of up to 10^4^ M^−1^ quenching constant was observed in the presence of both the common nitro-analytes (2,4-dinitrotoluene and 2,4,6-trinitrotoluene), while pentaerythritol tetranitrate was difficult to detect. In addition, the chemosensors reported in this study exhibited a dramatic fluorescence “turn-off” response to Hg^2+^ cation in aqueous solutions, compared to their response to other cations, with up to 10^4^ M^−1^ quenching constant and a limit of detection as low as 2.59 × 10^−6^ M. Based on both UV–Vis spectrophotometric titration studies and time-resolved fluorescence titration experiments, a false fluorescence static quenching mechanism was suggested for chemosensors **3a**-b in the presence of both the nitro-analytes and Hg^2+^ cation.

## Figures and Tables

**Table 2:** Values of Stern–Volmer constants of sensors **3a-b** and limits of detection of nitro-containing explosives.

Probes	Analyte	K_SV_, M^−1^	R^2^	LOD × 10^−7^, M
**3a**	DNT	0.597 × 10^4^	0.984	13.37
	TNT	0.712 × 10^4^	0.995	10.46
	PETN	0.418 × 10^4^	0.973	4.16
**3b**	DNT	0.772 × 10^4^	0.992	15.46
	TNT	0.767 × 10^4^	0.995	10.97
	PETN	0.468 × 10^4^	0.992	3.04

**Table 3:** Change of the distance between NO_2_- and methylene moiety for the sample **3a** (using the oxygen atom and hydrogen atom as a reference, d1_H–O_), between NO_2_- and triazole (using the oxygen atom and hydrogen atom as a reference, d2_H–O_), and between NO_2_- and phenyl moiety for **3b** (using the oxygen atom and hydrogen atom as a reference, d3_H–O_).

Probes	Distance, Å	DNT	TNT	PETN
**3a**	d1_H–O_	2.3	2.5	2.3
**3b**	d2_H–O_	3.5	2.2	-
	d3_H–O_	2.2	3.5	2.1
	d1_H–O_	-	-	2.3

**Table 4:** Calculated Gibbs free energies (G) for model structures in S_0_ state.

Model Structure	G, Hartree
TNT	−884.593347
DNT	−680.175562
PETN	−1015.221999
**3a**	−2503.482074
**3b**	−2657.178013
**3a**···TNT	−3388.042003
**3a**···DNT	−3183.635874
**3a**···PETN	−3518.681266
**3b**···TNT	−3541.754970
**3b**···DNT	−3337.325579
**3b**···PETN	−3672.378507

**Table 5:** HOMO/LUMO, energy gaps, and dipole moments in the ground state based on functionality of B3LYP_631pGs in gas phase.

Compound	HOMO, eV	LUMO, eV	ΔE, eV	µ_0_, D
**3a**	−6.15	−2.35	3.80	3.00
**3b**	−6.16	−2.36	3.80	3.06
DNT	−7.80	−3.00	4.80	3.08
TNT	−8.31	−3.39	4.92	1.76
PETN	−8.73	−2.91	5.82	0.0033

**Table 6:** Values of Stern–Volmer constants of sensors 3a-b and limits of detection of Hg^2+^ cation.

Probes	K_SV_, M^−1^	R^2^	LOD, M
**3a**	0.771 × 10^4^	0.966	2.59 × 10^−6^
**3b**	0.155 × 10^4^	0.991	1.86 × 10^−4^

## Data Availability

Data are contained within article.

## Supplementary Materials

The following supporting information can be downloaded at: https://www.mdpi.com/article/10.3390/molecules28135256/s1, Table S1: Lifetime measurement of probes **3a**,**b** in THF at r.t. (C = 2 × 10^−6^ M); Table S2: Data of wavelength of absorption/emission and Stokes shift in different solutions; Table S3: Lifetime measurement of probe **3b** in the presence of TNT in MeCN at r.t. (C = 1 × 10^−6^ M); Table S4: Lifetime measurement of probe **3a** in the presence of Hg^2+^ in MeCN:H_2_O [90:10 (vol.%)] at r.t. (C = 1 × 10^−6^ M); Table S5: Imaging of HOMO/LUMO of **3a**,**b** and analytes based on B3LYP/631pGs functional in gas phase; Table S6: The oscillator strengths for S_0_ → S_1_ and S_0_ → S_2_ transitions; Figure S1: ^1^H NMR (400 MHz, CDCl_3_) of **3a**; Figure S2: ^1^H NMR (400 MHz, CDCl_3_) of **3b**; Figure S3: Overlayed emission spectra for compound **3a** in the presence of DNT. Sample preparation: C(3a) = 1 × 10^−6^ M, λ_ex_ = 300 nm; Figure S4: Stern–Volmer plot for compound **3a** in the presence of DNT. Sample preparation: C(3a) = 1 × 10^−6^ M, λ_em_ = 350 nm; Figure S5: Limit of detection (LOD) plot for compound **3a** in the presence of DNT. Sample preparation: C(3a) = 1 × 10^−6^ M, λ_em_ = 350 nm; Figure S6: Overlayed emission spectra for compound **3a** in the presence of TNT. Sample preparation: C(3a) = 1 × 10^−6^ M, λ_ex_ = 300 nm; Figure S7: Stern–Volmer plot for compound **3a** in the presence of TNT. Sample preparation: C(3a) = 1 × 10^−6^ M, λ_em_ = 350 nm; Figure S8: Limit of detection (LOD) plot for compound **3a** in the presence of TNT. Sample preparation: C(3a) = 1 × 10^−6^ M, λ_em_ = 350 nm; Figure S9: Overlayed emission spectra for compound **3a** in the presence of PETN. Sample preparation: C(3a) = 1 × 10^−6^ M, λ_ex_ = 300 nm; Figure S10: Stern–Volmer plot for compound **3a** in the presence of PETN. Sample preparation: C(3a) = 1 × 10^−6^ M, λ_em_ = 350 nm; Figure S11: Limit of detection (LOD) plot for compound **3a** in the presence of PETN. Sample preparation: C(3a) = 1 × 10^−6^ M, λ_em_ = 350 nm; Figure S12: Overlayed emission spectra for compound **3a** in the presence of Hg^2+^. Sample preparation: C(3a) = 1 × 10^−6^ M, λ_ex_ = 300 nm; Figure S13: Stern–Volmer plot for compound **3a** in the presence of Hg^2+^. Sample preparation: C(3a) = 1 × 10^−6^ M, λ_em_ = 350 nm; Figure S14: Limit of detection (LOD) plot for compound **3a** in the presence of Hg^2+^. Sample preparation: C(3a) = 1 × 10^−6^ M, λ_em_ = 350 nm; Figure S15: Overlayed emission spectra for compound **3b** in the presence of DNT. Sample preparation: C(3b) = 1 × 10^−6^ M, λ_ex_ = 296 nm; Figure S16: Stern–Volmer plot for compound **3b** in the presence of DNT. Sample preparation: C(3b) = 1 × 10^−6^ M, λ_em_ = 350 nm; Figure S17: Limit of detection (LOD) plot for compound **3b** in the presence of DNT. Sample preparation: C(3b) = 1 × 10^−6^ M, λ_em_ = 350 nm; Figure S18: Overlayed emission spectra for compound **3b** in the presence of TNT. Sample preparation: C(3b) = 1 × 10^−6^ M, λ_ex_ = 296 nm; Figure S19: Stern–Volmer plot for compound **3b** in the presence of TNT. Sample preparation: C(3b) = 1 × 10^−6^ M, λ_em_ = 350 nm; Figure S20: Limit of detection (LOD) plot for compound **3b** in the presence of TNT. Sample preparation: C(3b) = 1 × 10^−6^ M, λ_em_ = 350 nm; Figure S21: Overlayed emission spectra for compound **3b** in the presence of PETN. Sample preparation: C(3b) = 1 × 10^−6^ M, λ_ex_ = 296 nm; Figure S22: Stern–Volmer plot for compound **3b** in the presence of PETN. Sample preparation: C(3b) = 1 × 10^−6^ M, λ_em_ = 350 nm; Figure S23 Limit of detection (LOD) plot for compound **3b** in the presence of PETN. Sample preparation: C(3b) = 1 × 10^−6^ M, λ_em_ = 350 nm; Figure S24: Overlayed emission spectra for compound **3b** in the presence of Hg^2+^. Sample preparation: C(3b) = 1 × 10^−6^ M, λ_ex_ = 296 nm; Figure S25 Stern–Volmer plot for compound **3b** in the presence of Hg^2+^. Sample preparation: C(3b) = 1 × 10^−6^ M, λ_em_ = 350 nm; Figure S26 Limit of detection (LOD) plot for compound **3b** in the presence of Hg^2+^. Sample preparation: C(3b) = 1 × 10^−6^ M, λ_em_ = 350 nm; Figure S27: Overlayed absorption spectra for compound **3b** in the presence of TNT. Sample preparation: C(3b) = 1 × 10^−6^ M; Figure S28: Overlayed time-resolved emission spectra for compound **3b** in the presence of TNT. Sample preparation: C(3b) = 1 × 10^−6^ M; Figure S29: The graphical result of time-resolved fluorescence titration of **3b** by TNT monitored at 350 nm; Figure S30: Overlayed absorption spectra for compound **3a** in the presence of Hg^2+^. Sample preparation: C(3a) = 1 × 10^−6^ M; Figure S31: Overlayed time-resolved emission spectra for compound **3a** in the presence of Hg^2+^. Sample preparation: C(3a) = 1 × 10^−6^ M; Figure S32: The graphical result of time-resolved fluorescence titration of **3a** by Hg^2+^ monitored at 350 nm; Figure S33: Geometric structures of the possible configurations for probes 3a,b combining with molecular of analyte DNT/TNT/PETN (adducts): 3a_DNT, 3a_TNT, 3a_PETN, 3b_DNT, 3b_TNT, 3b_PETN on ground state; Figure S34: Qualitative assessment of the presence of metal ions by adding to the MeCN:H_2_O [90:10 (vol.%)] solution of probes **3a** (upper row) and **3b** (bottom row) (2 × 10^−4^M) aqueous solution of salt (2 × 10^−2^M, ~1.0 eq.) under 365 nm UV light.

## Sample Availability

Samples of the compounds **3a-b** are available from the authors.
