# Supplementary material for: (1-(4-(5-Phenyl-1,3,4-oxadiazol-2-yl)phenyl)-1H-1,2,3-triazol-4-yl)-methylenyls α,ω-Bisfunctionalized 3- and 4-PEG: Synthesis and Photophysical Studies
Source: Molecules. 2023 Jul 6;28(13):5256. doi: 10.3390/molecules28135256 (PMC10343605; doi:10.3390/molecules28135256)
Supplement: Supplementary file 1 [file molecules-28-05256-s001.zip › molecules-2456493-supplementary.pdf]

Article

(1-(4-(5-Phenyl-1,3,4-oxadiazol-2-yl)phenyl)-1*H*-1,2,3-triazol-4-yl)-methylenyls α,ω-Bisfunctionalized 3- and 4-PEG: Synthesis and Photophysical Studies

Mohammed S. Mohammed [1], Igor S. Kovalev [1], Natalya V. Slovesnova [1,2], Leila K. Sadieva [1,*], Vadim A. Platonov [1], Grigory A. Kim [3,4], Rammohan Aluru [1], Alexander S. Novikov [5,6], Olga S. Taniya [1] and Valery N. Charushin [1,3]

[1] Chemical Engineering Institute, Ural Federal University, 19 Mira St., 620002 Yekaterinburg, Russia; mmokhammed@urfu.ru (M.S.M.); i.s.kovalev@urfu.ru (I.S.K.); n.v.slovesnova@urfu.ru (N.V.S.); vadim.platonov@urfu.ru (V.A.P.); raluru@urfu.ru (R.A.); olga.tania@urfu.ru (O.S.T.); v.n.charushin@urfu.ru (V.N.C.)

[2] Department of Pharmacy and Chemistry, Ural Medical University, 3 Repina St., 620028 Yekaterinburg, Russia

[3] I. Ya. Postovsky Institute of Organic Synthesis of RAS (Ural Division), 22/20, S. Kovalevskoy/Akademicheskaya St., 620137 Yekaterinburg, Russia; kim-g@ios.uran.ru

[4] Institute of Natural Sciences and Mathematics, Ural Federal University, 19 Mira St., 620002 Yekaterinburg, Russia

[5] Institute of Chemistry, Saint Petersburg State University, Universitetskaya Nab., 7/9, 199034 Saint Petersburg, Russia; a.s.novikov@spbu.ru

[6] Research Institute of Chemistry, Peoples' Friendship University of Russia (RUDN University), Miklukho-Maklaya Street, 6, 117198 Moscow, Russia

* Correspondence: l.k.sadieva@urfu.ru

Contents

1. Materials and equipment 3
2. Experimental procedures 3
3. NMR spectra 4
4. Optical studies 5
5. Stern-Volmer calculations 6
6. Additional studies of mechanism of quenching 18
7. DFT calculations 22
8. Recognition of Hg^{2+} via fluorescence "turn off" process 25

1. Materials and equipment

Unless otherwise indicated, all common reagents and solvents were used from commercial suppliers without further purification. Melting points were determined on Boetius combined heating stages. TLC and column chromatography were carried out on SiO_2. ^{1}H NMR and ^{13}C NMR spectra were recorded at room temperature at 400 and 100 MHz respectively, on a Bruker DRX-400 spectrometer (Bruker BioSpin GmbH, Rheinstetten, Germany) using $CDCl_3$ as the solvent. Peaks were labeled as singlet (s), doublet (d), triplet (t), doublet of doublets (dd), doublet of doublets of doublets (ddd) and multiplet (m). The mass spectra (electron impact) were measured on a Shimadzu GCMS-QP2010 Ultra (Shimadzu, Kyoto, Japan) instrument ionization. Elemental analyses were performed on a PerkinElmer 2400 Series II CHN-analyzer (PerkinElmer, Waltham, USA). UV-Vis absorption spectra were recorded on the Shimadzu UV-1800 spectrophotometer (Shimadzu, Kyoto, Japan), and emission spectra were measured on the Horiba FluoroMax-4 (HORIBA Jobin Yvon S.A.S., Longjumeau, France) by using quartz cells with 1 cm path length at room temperature. Absolute quantum yields of luminescence of target compounds in solution were measured by using the Integrating Sphere Quanta-φ of the Horiba-Fluoromax-4 (HORIBA Jobin Yvon S.A.S., Longjumeau, France) at room temperature. The fluorometric titration was performed by the single-point methodology using Horiba FluoroMax-4 (HORIBA Jobin Yvon S.A.S., Longjumeau, France).

2. Experimental procedures

In 50 mL ace flask were successively added corresponding 2- (4-azidophenyl) -5-aryl-1,3,4-oxadiazole (2.20 eqv.), copper(I) iodide (0.20 eqv.), ethynyl compound (1 eqv.) were added to 5 mL of in THF:H_2O [90:10 (vol.%)]. RM heated for 10 h at 65 °C in an argon atmosphere. After the reaction was completed (TLC monitoring), the reaction mass was diluted by aqueous 10% NH_4OH (10 mL). The resulting suspension was filtered. Cake dried on air. Product was purified by flash chromatography if needed.

1,12-bis(1-(4-(5-phenyl-1,3,4-oxadiazol-2-yl)phenyl)-1*H*-1,2,3-triazol-4-yl)-2,5,8,11-tetraoxadodecane (3a). Yield 103 mg, 30%. ^{1}H NMR in $CDCl_3$, ppm: 3.71 (s, 4H, 2×CH_2O), 3.73- 3.76 (m, 4H, 2×CH_2O), 3.77- 3.81 (m, 4H, 2×CH_2O), 4.82 (s, 4H, 2×CH_2), 7.54–7.59 (m, 6H, C_6H_5), 7.96 (m, 4H, C_6H_4), 8.15 (m, 4H, C_6H_5), 8.21 (s, 2H, C_2N_3H), 8.28 (m, 4H, C_6H_4). ^{13}C NMR in DMSO-d_6, ppm: 63.2 (1C), 69.0 (1C), 69.6 (1C), 70 (1C), 120.3 (1C), 122 (1C), 123 (1C), 123.1 (1C), 126.6 (1C), 128.1 (1C), 129.2 (1C), 132 (1C), 138.5 (1C), 145.4 (1C), 163 (1C), 164 (1C). EI-MS, m/z (I, %): 753 (1). Fnd, %: C 63.73, H 4.95, N 18.54; calc. for $C_{40}H_{36}N_{10}O_6$, %: C 63.82, H 4.82, N, 18.61.

1,15-bis(1-(4-(5-phenyl-1,3,4-oxadiazol-2-yl)phenyl)-1*H*-1,2,3-triazol-4-yl)-2,5,8,11,14-pentaoxapentadecane (3b). Yield 311 mg, 75%. ^{1}H NMR in $CDCl_3$, ppm: 3.69 (s, 8H, 4×CH_2O), 3.70- 3.73 (m, 4H, 2×CH_2O), 3.75- 3.79 (m, 4H, 2×CH_2O), 4.80 (s, 4H, 2×CH_2), 7.54–7.59 (m, 6H, C_6H_5), 7.97 (m, 4H, C_6H_4), 8.13- 8.18 (m, 4H, C_6H_5), 8.23 (s, 2H, C_2N_3H), 8.29 (m, 4H, C_6H_4). ^{13}C NMR in DMSO-*d6*, ppm: 64 (1C), 70 (1C), 70.2 (1C), 70.3 (1C), 121 (1C), 122.6 (1C), 123.6 (1C), 124 (1C), 127.2 (1C), 129 (1C), 130 (1C), 132.5 (1C), 139.3 (1C), 146 (1C), 164 (1C), 165 (1C). EI-MS, m/z (I, %): $[M-C_{17}H_{12}N_5O_2]^+$ = 318 (6). Fnd, %: C 63.24, H 5.10, N 17.69; calc. for $C_{42}H_{40}N_{10}O_7$, %: C 63.31, H 5.06, N 17.58.

3. NMR spectra

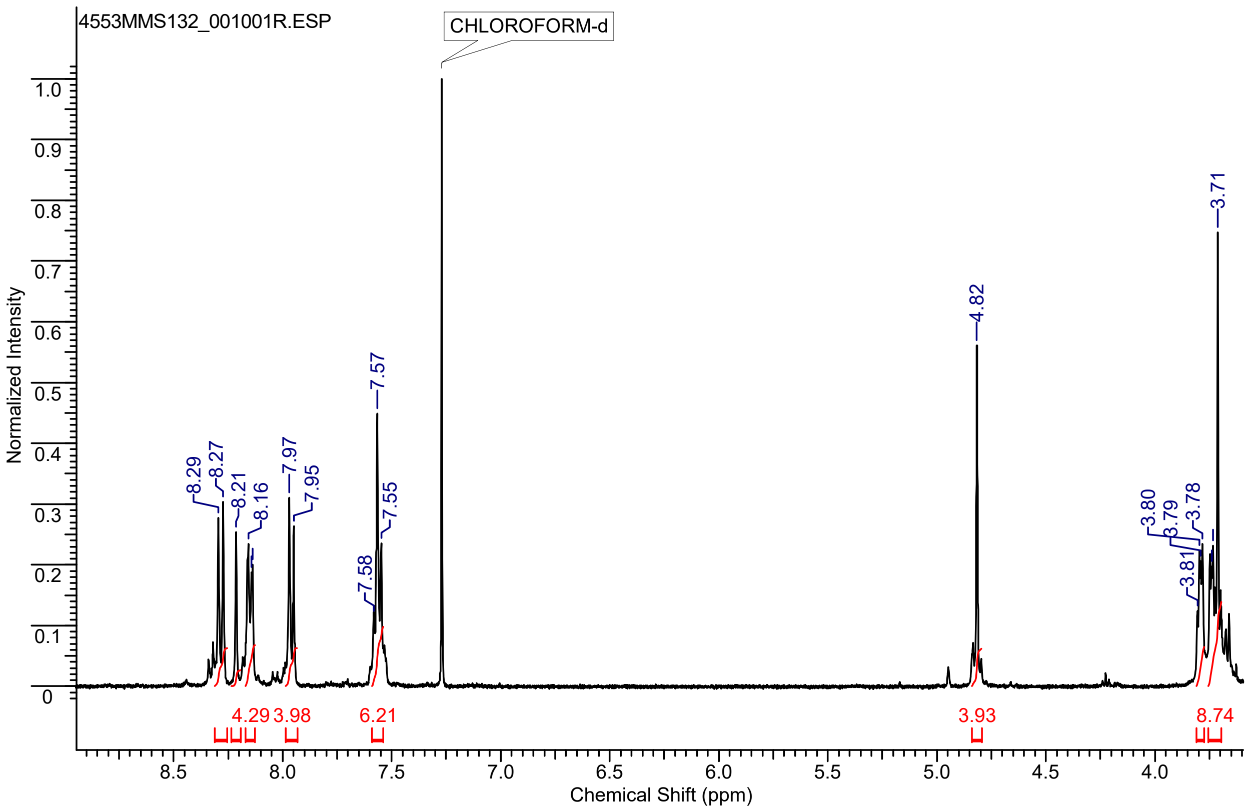

Figure S1. ^{1}H NMR (400 MHz, $CDCl_3$) of **3a.**

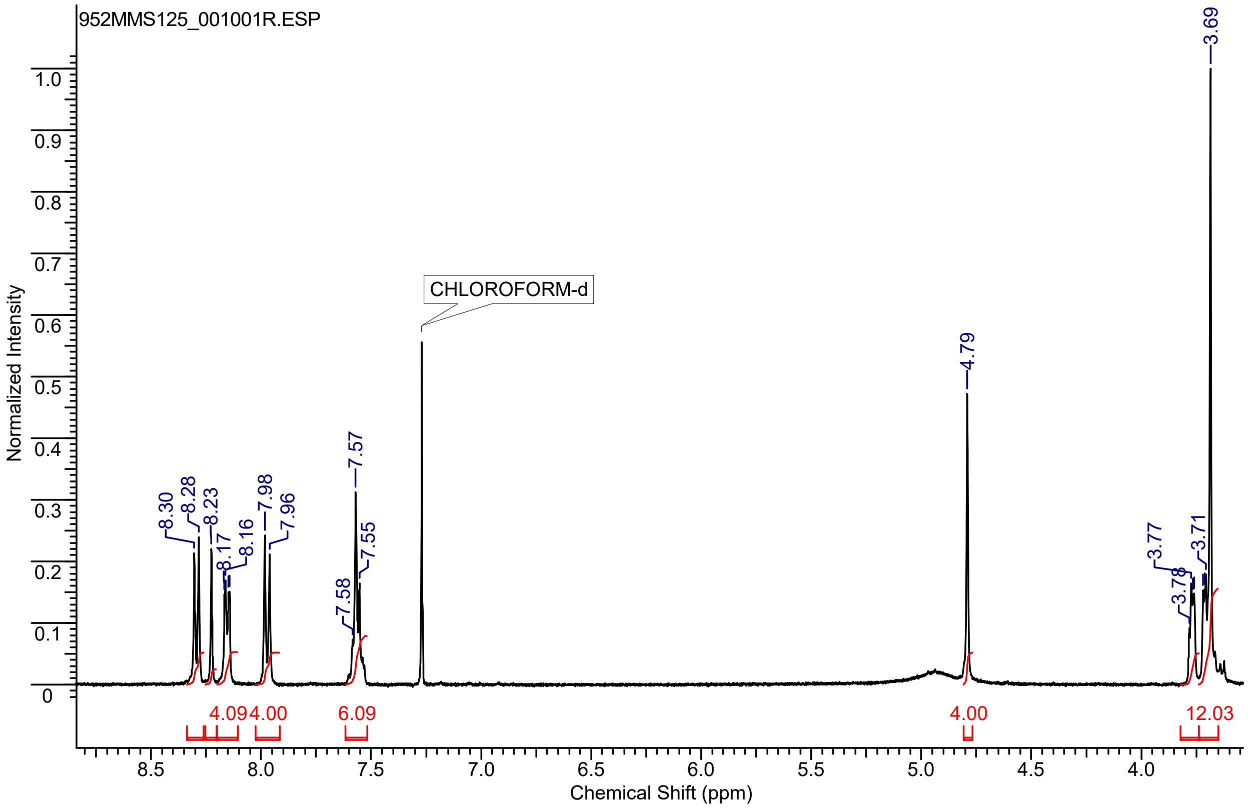

Figure S2. ^{1}H NMR (400 MHz, $CDCl_3$) of **3b.**

4. Optical Studies

Table S1. Lifetime measurement of probes **3a,b** in THF at r.t. (C = 2×10^{-6} M).

Entry	Compound	τ_1, ns[a]	α_1[b]	τ_2, ns[a]	α_2[b]	τ_{av}, ns[a]	χ^{2}[d]
**1**	**3a**	0.94	*0.84*	1.80	0.16	3.4	1.11
**2**	**3b**	0.69	*0.28*	1.52	0.72	2.6	1.10

[a] Decay time, [b] Fractional contribution, [c] Weighted average decay time $\tau_{av} = \Sigma (\tau_i \times \alpha_i)$, [d] Quality of fitting.

Table S2. Data of wavelength of absorption/emission and Stokes shift in different solutions.

3a	$\lambda_{abs\ max}$, nm	$\lambda_{em\ max}$, nm	Stokes shift, nm	3b	$\lambda_{abs\ max}$, nm	$\lambda_{em\ max}$, nm	Stokes shift, nm
**DMSO**	301	337<br>354<br>366	65	**DMSO**	304	337<br>354<br>366	62
**MeCN**	296	333<br>349<br>365	69	**MeCN**	296	332<br>349<br>369	75
**DCM**	298	335<br>351<br>364	66	**DCM**	299	332<br>349<br>368	69
**MeOH**	296	334<br>351<br>364	68	**MeOH**	296	335<br>350<br>364	68
**THF**	299	335<br>350<br>364	65	**THF**	300	335<br>350<br>364	64

5. Stern-Volmer Calculations

The Stern-Volmer quenching model show the dependence of the change in the emission intensity on the analyte concentration is determined.

$$I_0/I = 1 + K_{sv}\,[Q] \quad (1)$$

The coupling constant for the Stern-Volmer equation was calculated as the tangent of the slope of the dependence plot $((I_0/I)-1)$ on the concentration of the quencher ([Q]). The analyte concentration [Q] was recalculated using the formula: $[Q] = (C_q \times V_q)/(V_q+3E\text{-}3)$.

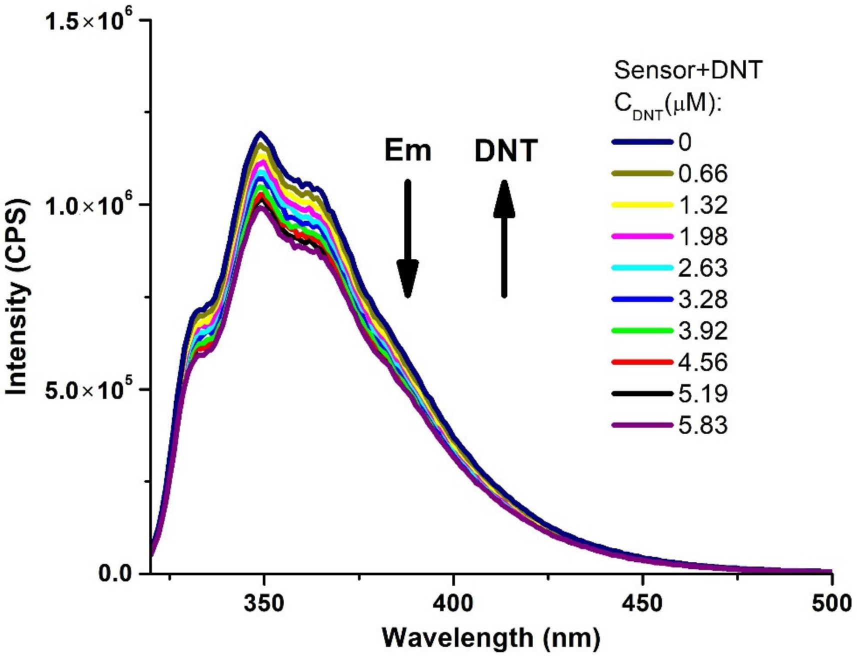

Figure S3. Overlayed emission spectra for compound **3a** in the presence of DNT. Sample preparation: C(3a) = 1 × 10^{-6} M, λ_{ex} = 300 nm.

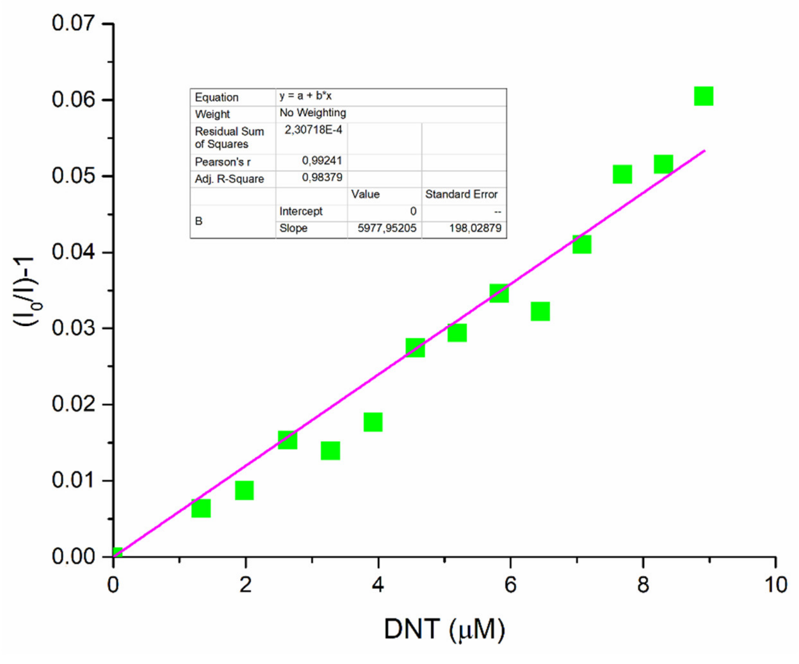

Figure S4. Stern-Volmer plot for compound **3a** in the presence of DNT. Sample preparation: C(3a) = 1 × 10^{-6} M, λ_{em} = 350 nm.

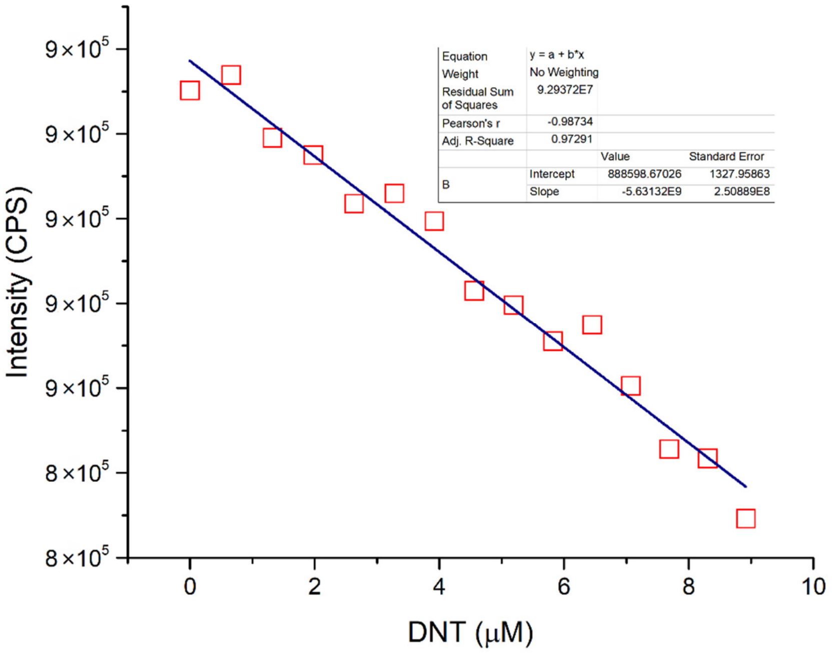

Figure S5. Limit of detection (LOD) plot for compound **3a** in the presence of DNT. Sample preparation: C(3a) = 1×10^{-6} M, λ_{em} = 350 nm.

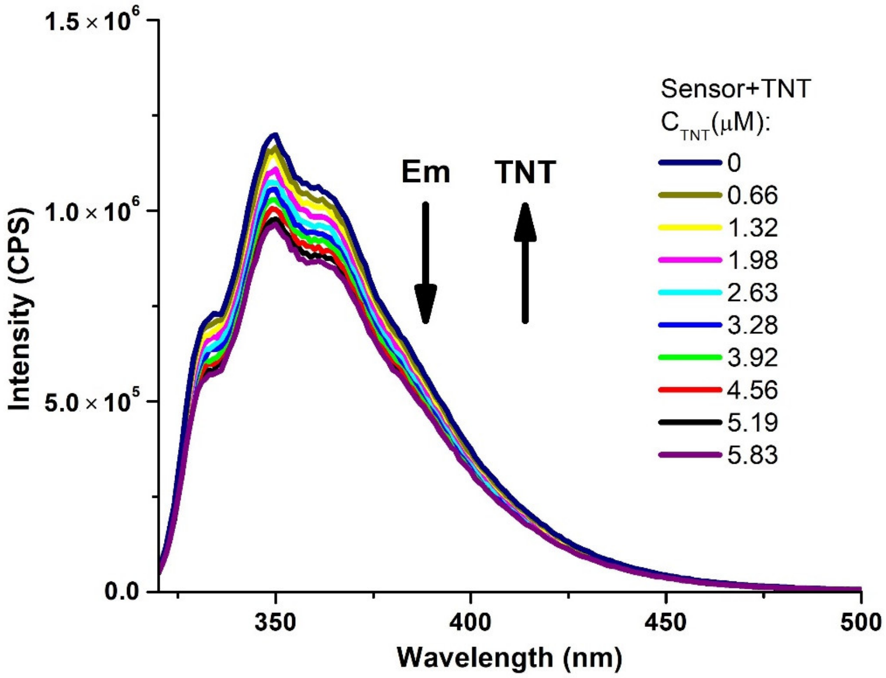

Figure S6. Overlayed emission spectra for compound **3a** in the presence of TNT. Sample preparation: C(3a) = 1×10^{-6} M, λ_{ex} = 300 nm.

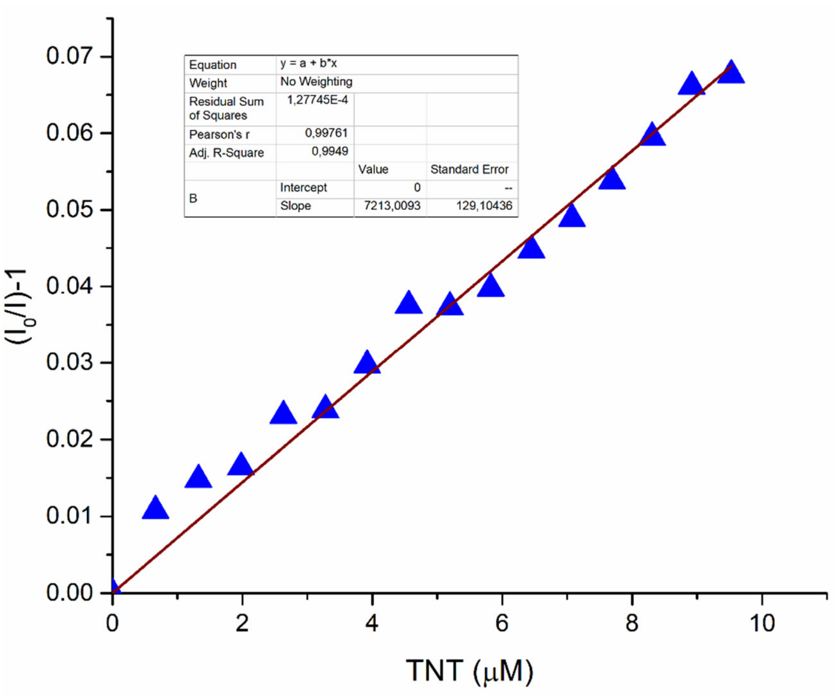

Figure S7. Stern-Volmer plot for compound **3a** in the presence of TNT. Sample preparation: C(3a) = $1{\times}10^{-6}$ M, λ_{em} = 350 nm.

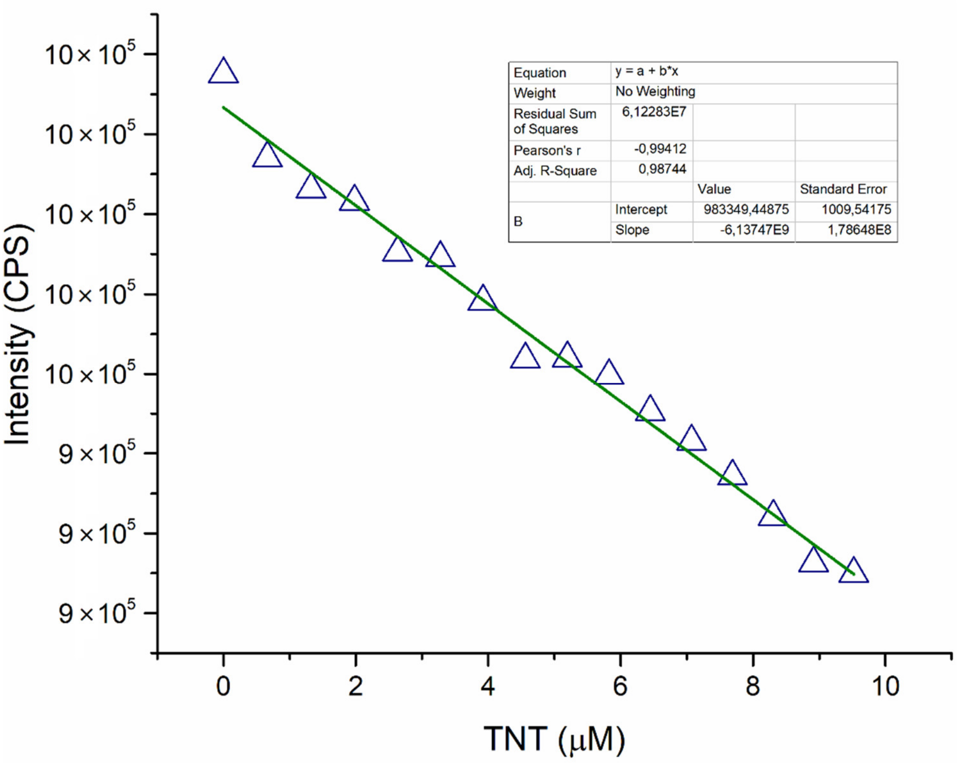

Figure S8. Limit of detection (LOD) plot for compound **3a** in the presence of TNT. Sample preparation: C(3a) = $1{\times}10^{-6}$ M, λ_{em} = 350 nm.

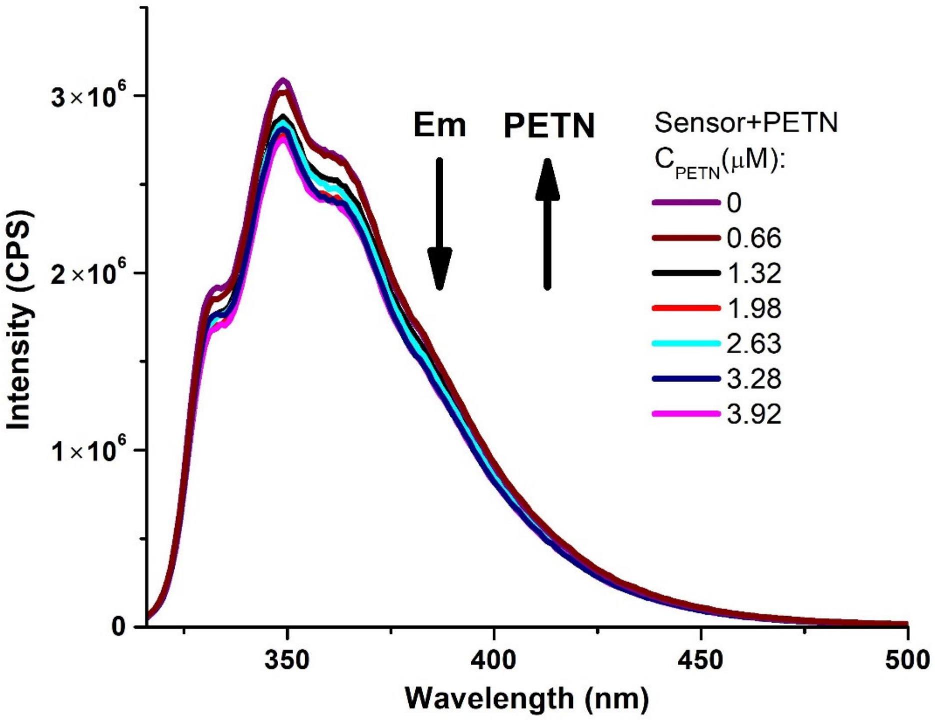

Figure S9. Overlayed emission spectra for compound **3a** in the presence of PETN. Sample preparation: C(3a) = 1×10^{-6} M, λ_{ex} = 300 nm.

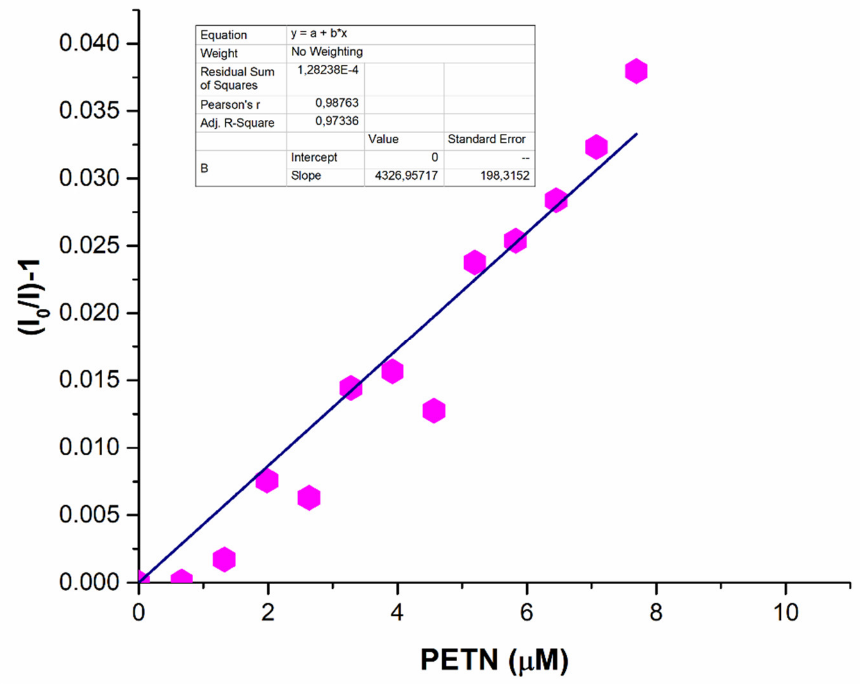

Figure S10. Stern-Volmer plot for compound **3a** in the presence of PETN. Sample preparation: C(3a) = 1×10^{-6} M, λ_{em} = 350 nm.

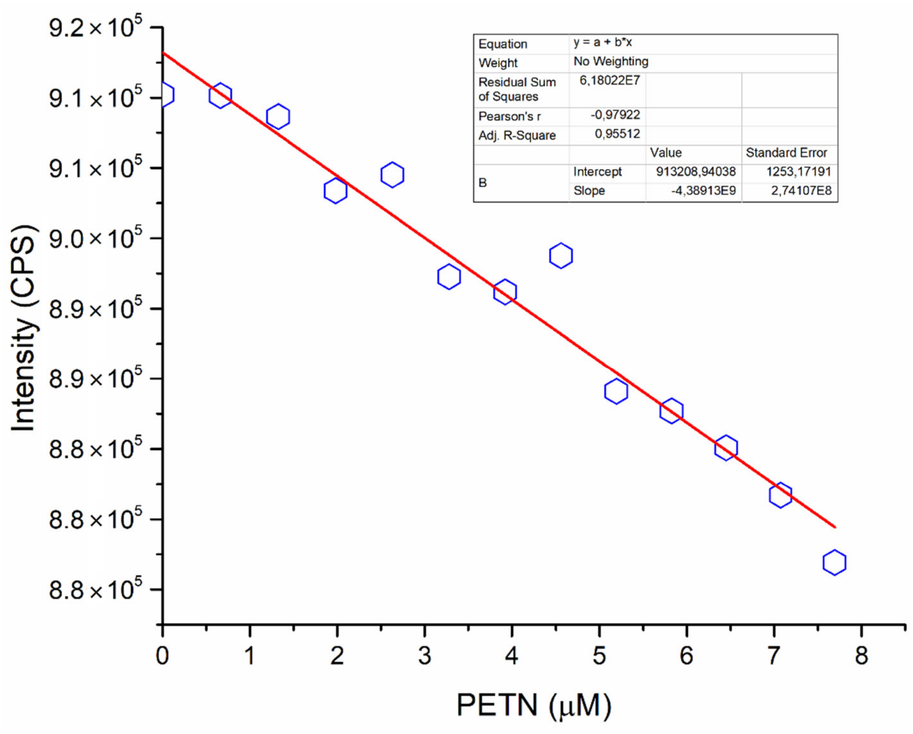

Figure S11. Limit of detection (LOD) plot for compound **3a** in the presence of PETN. Sample preparation: C(3a) = 1×10^{-6} M, λ_{em} = 350 nm.

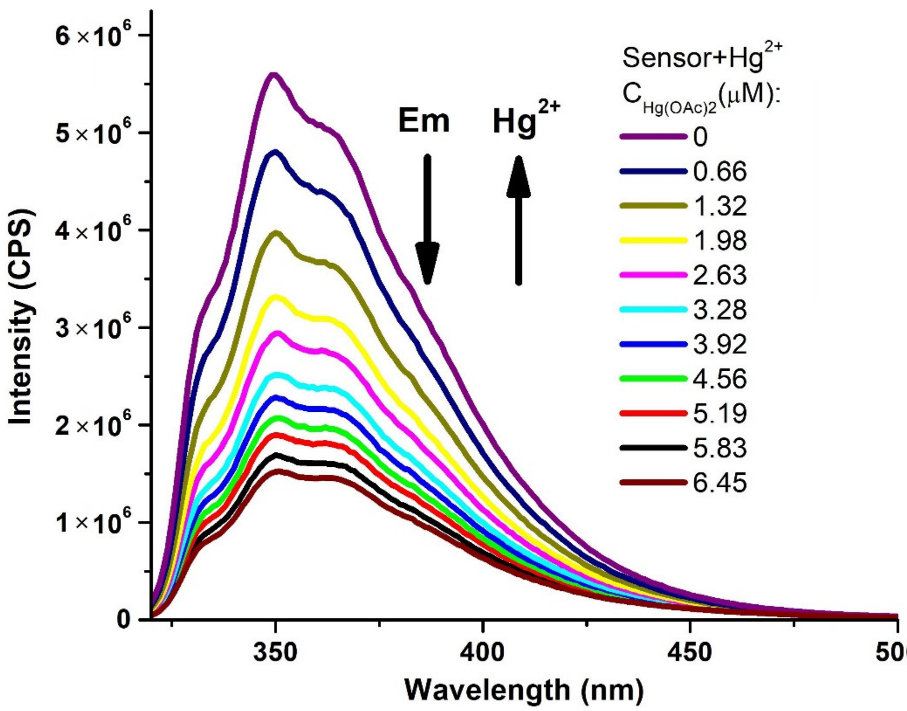

Figure S12. Overlayed emission spectra for compound **3a** in the presence of Hg^{2+}. Sample preparation: C(3a) = 1×10^{-6} M, λ_{ex} = 300 nm.

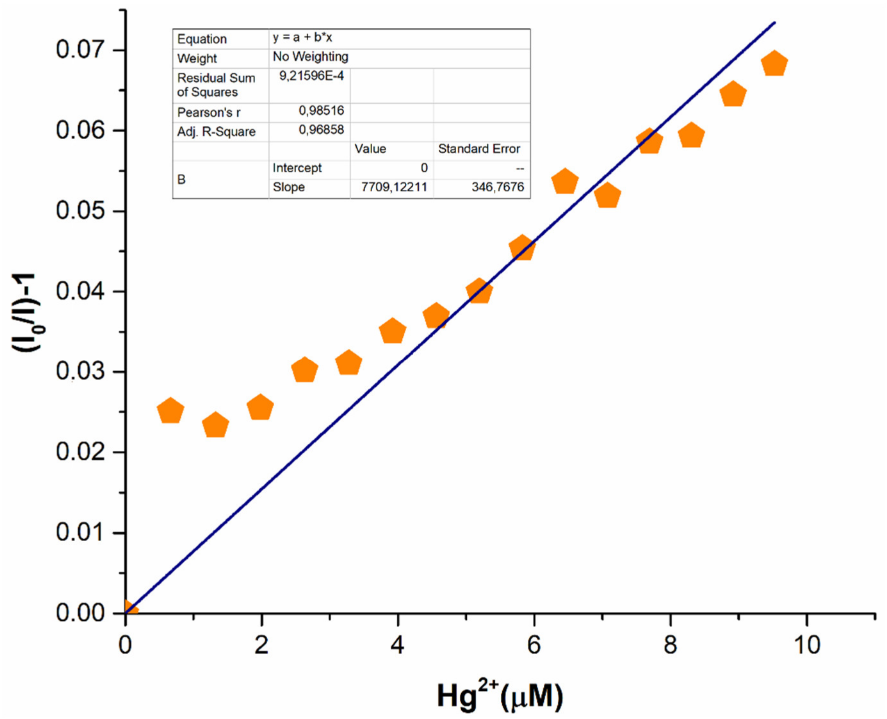

Figure S13. Stern-Volmer plot for compound **3a** in the presence of Hg^{2+}. Sample preparation: C(3a) = 1×10^{-6} M, λ_{em} = 350 nm.

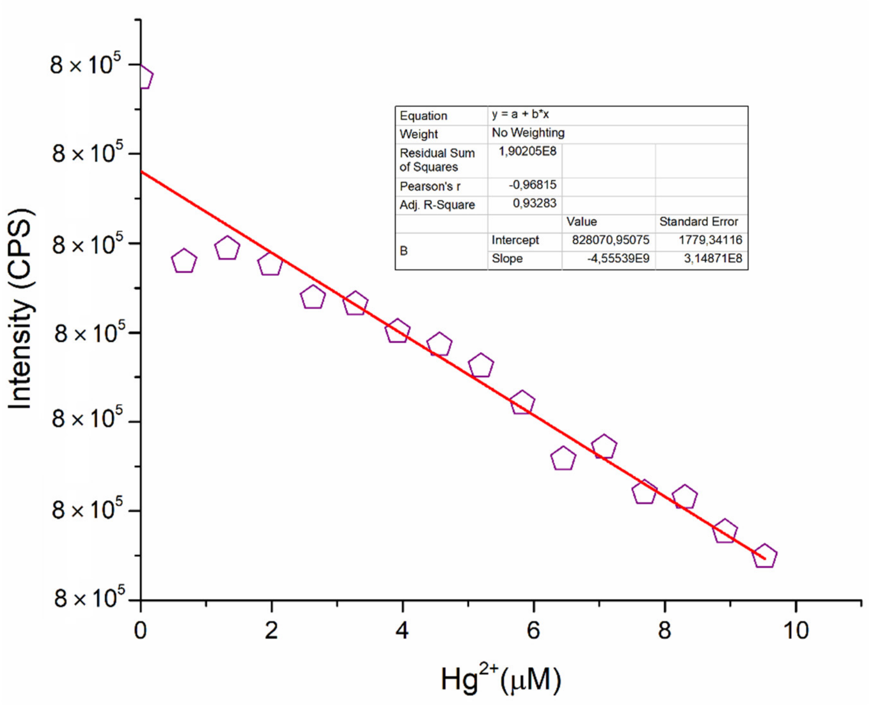

Figure S14. Limit of detection (LOD) plot for compound **3a** in the presence of Hg^{2+}. Sample preparation: C(3a) = 1×10^{-6} M, λ_{em} = 350 nm.

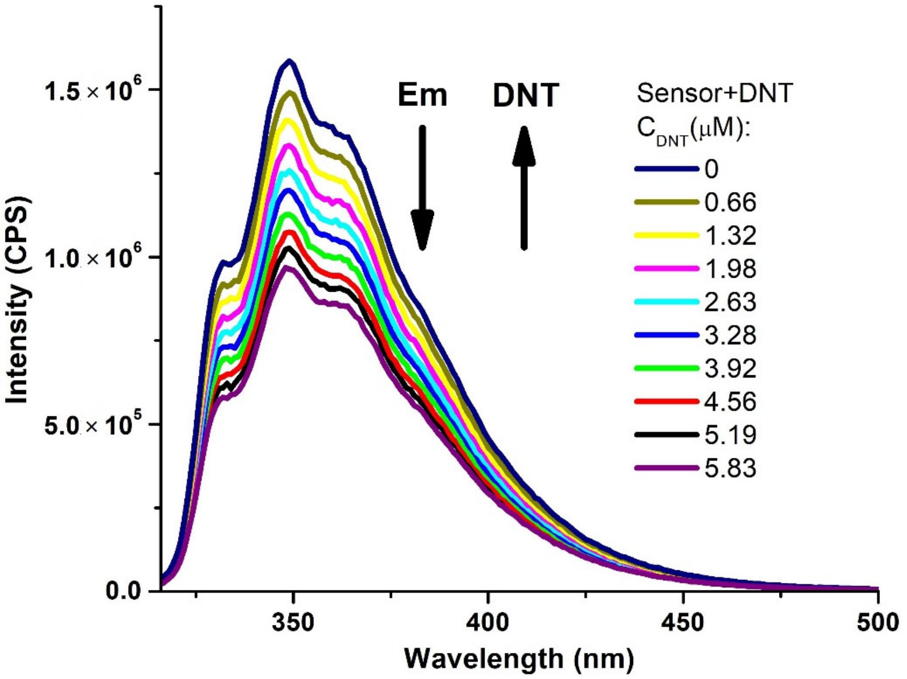

Figure S15. Overlayed emission spectra for compound **3b** in the presence of DNT. Sample preparation: C(3b) = 1×10^{-6} M, λ_{ex} = 296 nm.

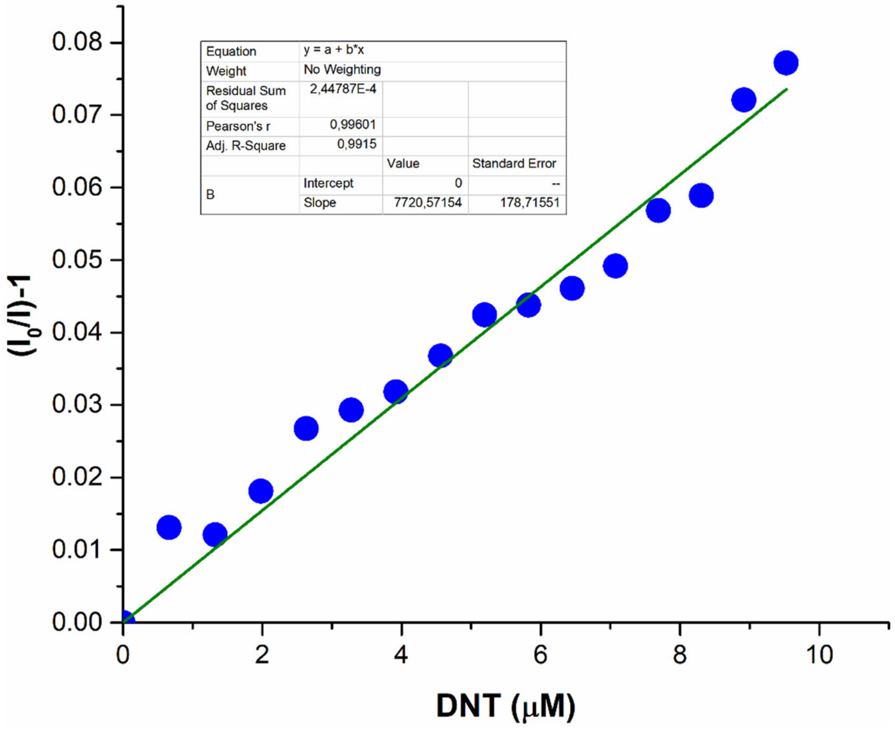

Figure S16. Stern-Volmer plot for compound **3b** in the presence of DNT. Sample preparation: C(3b) = 1×10^{-6} M, λ_{em} = 350 nm.

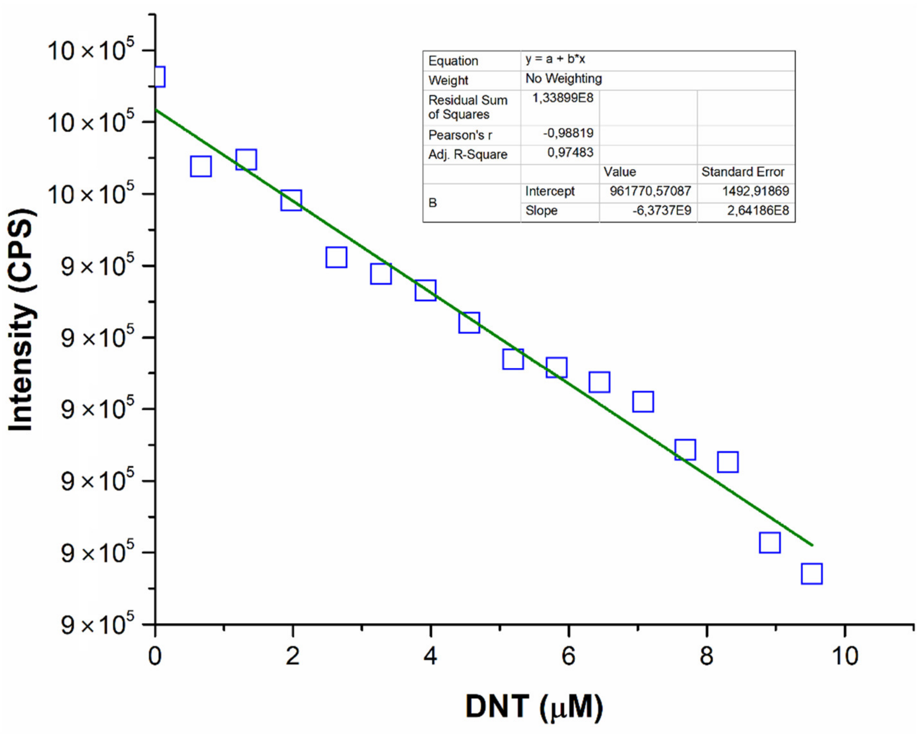

Figure S17. Limit of detection (LOD) plot for compound **3b** in the presence of DNT. Sample preparation: C(3b) = 1×10^{-6} M, λ_{em} = 350 nm.

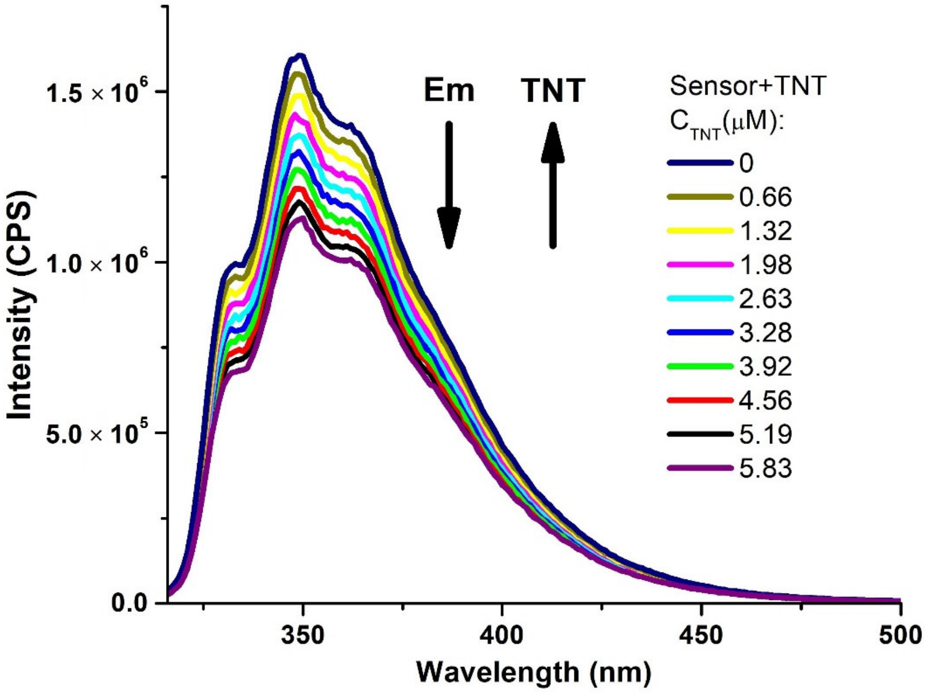

Figure S18. Overlayed emission spectra for compound **3b** in the presence of TNT. Sample preparation: C(3b) = 1×10^{-6} M, λ_{ex} = 296 nm.

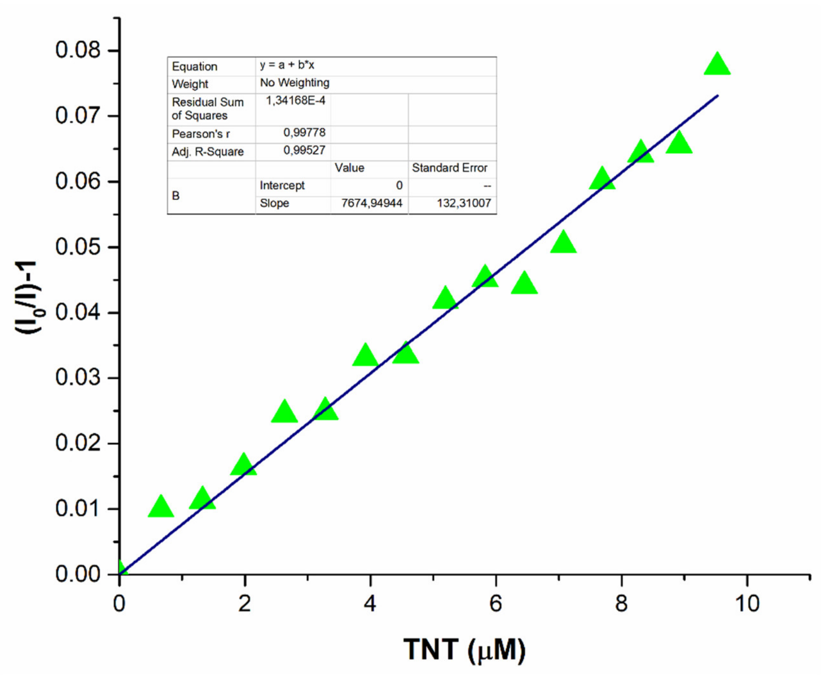

Figure S19. Stern-Volmer plot for compound **3b** in the presence of TNT. Sample preparation: C(3b) = 1×10^{-6} M, λ_{em} = 350 nm.

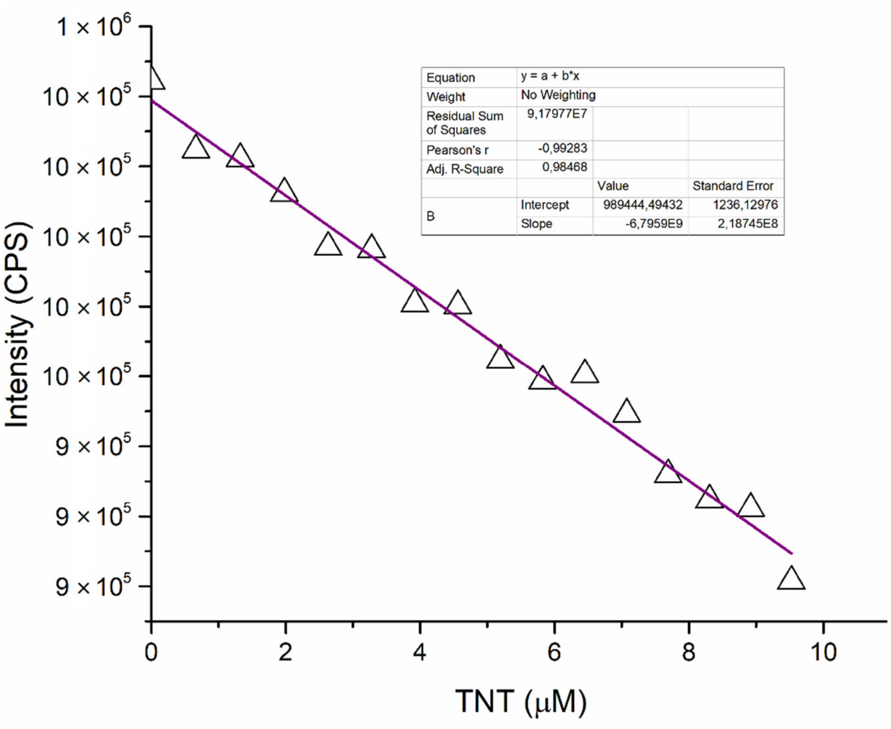

Figure S20. Limit of detection (LOD) plot for compound **3b** in the presence of TNT. Sample preparation: C(3b) = 1×10^{-6} M, λ_{em} = 350 nm.

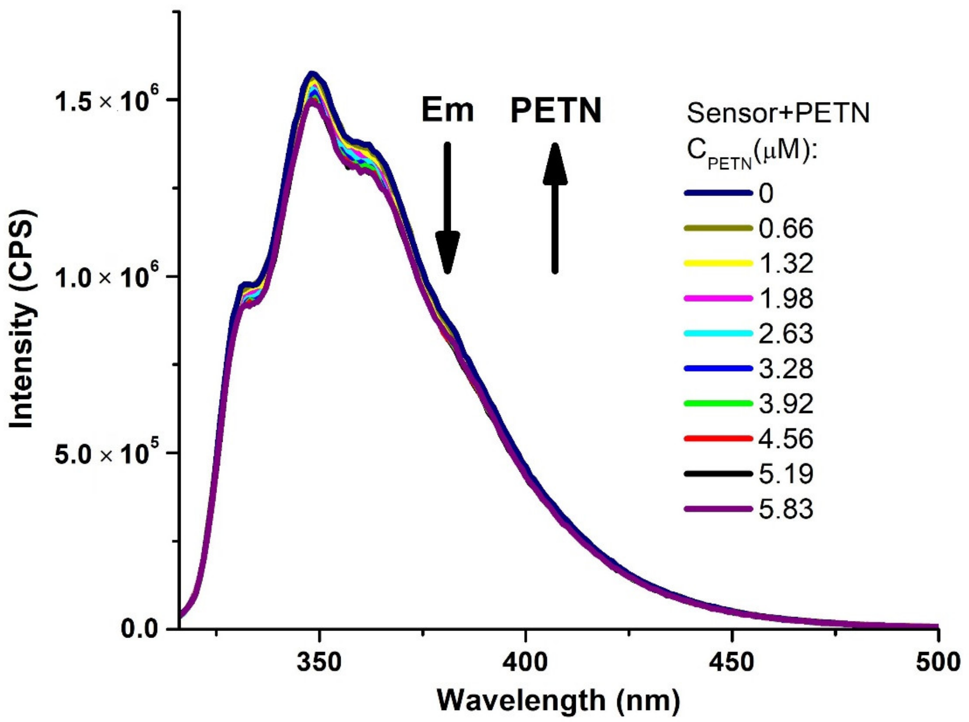

Figure S21. Overlayed emission spectra for compound **3b** in the presence of PETN. Sample preparation: C(3b) = 1×10^{-6} M, λ_{ex} = 296 nm.

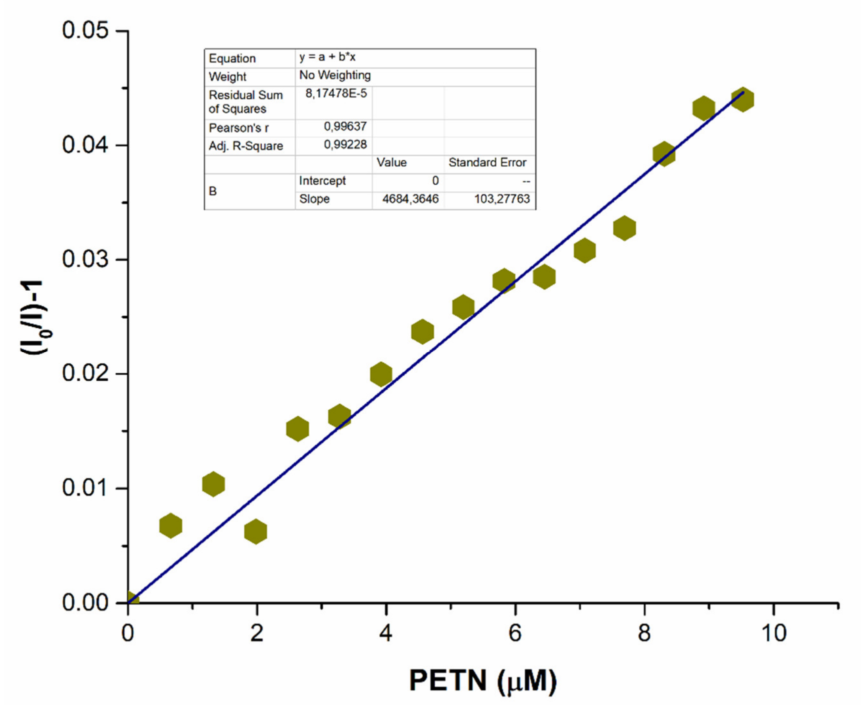

Figure S22. Stern-Volmer plot for compound **3b** in the presence of PETN. Sample preparation: C(3b) = 1×10^{-6} M, λ_{em} = 350 nm.

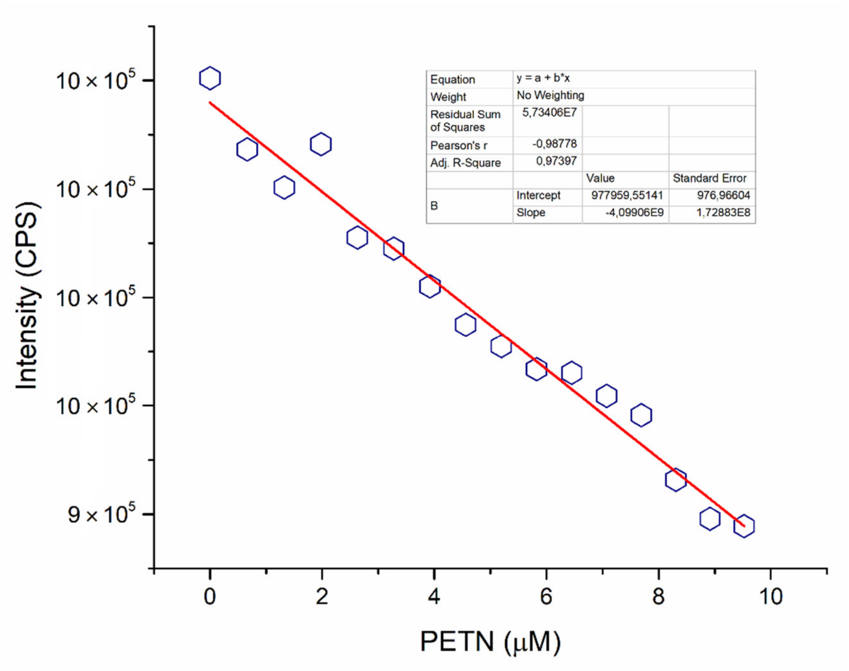

Figure S23. Limit of detection (LOD) plot for compound **3b** in the presence of PETN. Sample preparation: C(3b) = 1×10^{-6} M, λ_{em} = 350 nm.

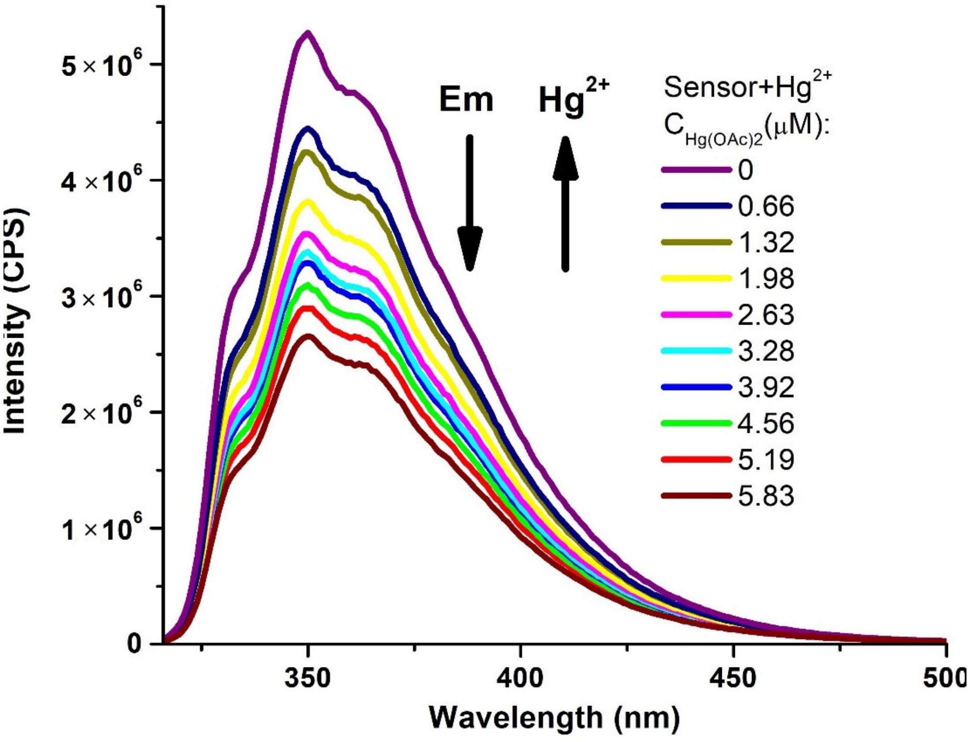

Figure S24. Overlayed emission spectra for compound **3b** in the presence of Hg^{2+}. Sample preparation: C(3b) = 1×10^{-6} M, λ_{ex} = 296 nm.

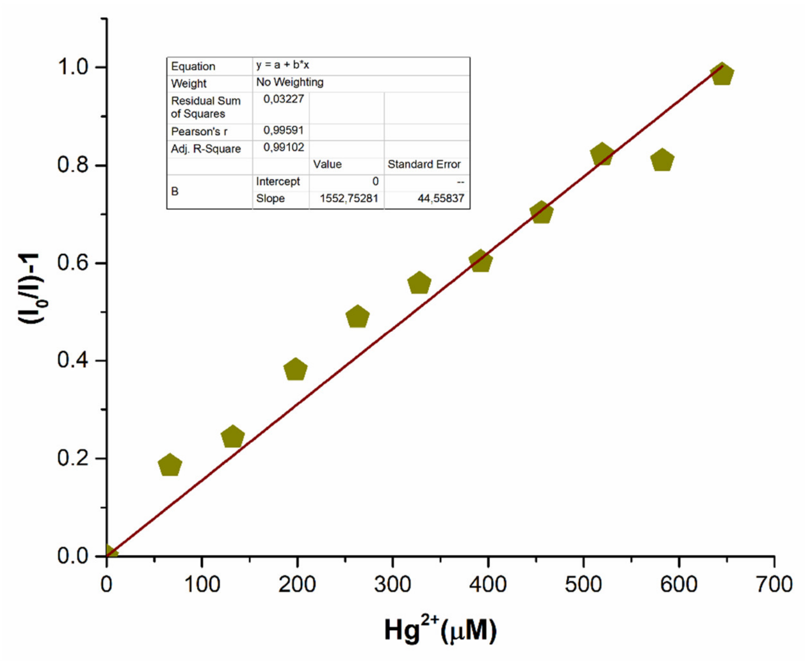

Figure S25. Stern-Volmer plot for compound **3b** in the presence of Hg^{2+}. Sample preparation: C(3b) = 1×10^{-6} M, λ_{em} = 350 nm.

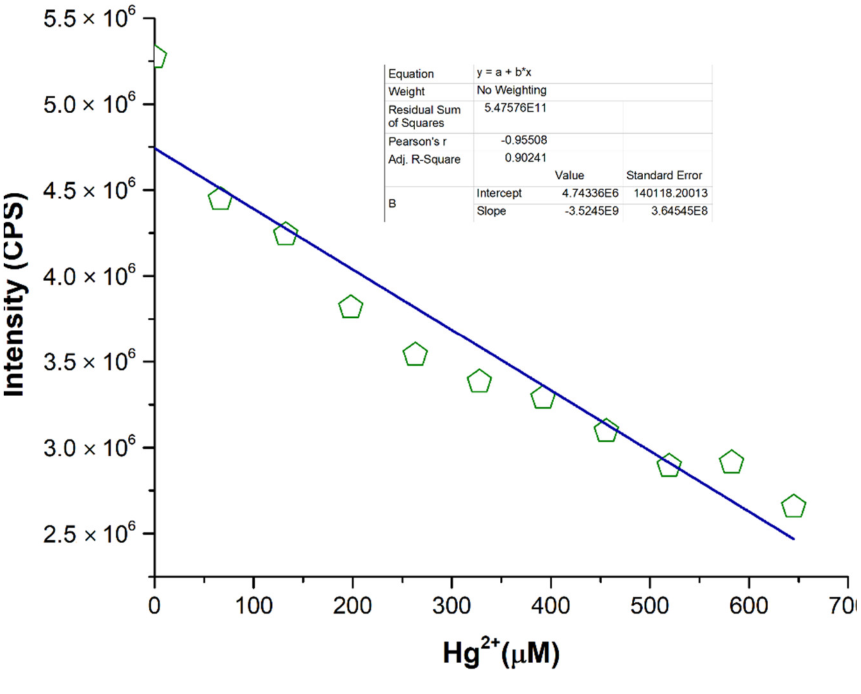

Figure S26. Limit of detection (LOD) plot for compound **3b** in the presence of Hg^{2+}. Sample preparation: C(3b) = 1×10^{-6} M, λ_{em} = 350 nm.

6. Additional Studies of Mechanism of Quenching

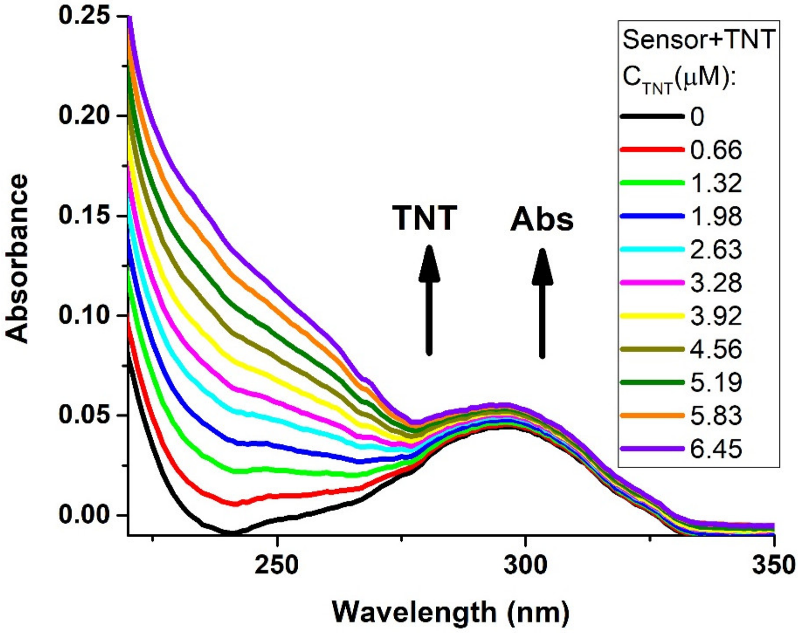

Figure S27. Overlayed absorption spectra for compound **3b** in the presence of TNT. Sample preparation: C(3b) = 1×10^{-6} M.

Table S3. Lifetime measurement of probe **3b** in the presence of TNT in MeCN at r.t. (C = 1×10^{-6} M).

Entry	C_{TNT}, μM	τ_1, ns [a]	α_1 [b]	τ_2, ns [a]	α_2 [b]	τ_{av}, ns [c]
**1**	0	0.67	*0.56*	1.87	0.44	1.19
**2**	0.66	0.68	*0.57*	1.87	0.43	1.19
**3**	1.32	0.64	*0.53*	1.81	0.47	1.19
**4**	1.98	0.67	*0.56*	1.86	0.44	1.19
**5**	2.63	0.64	*0.54*	1.84	0.46	1.19
**6**	3.28	0.67	*0.56*	1.86	0.44	1.19
**7**	3.92	0.65	*0.54*	1.83	0.46	1.19
**8**	4.56	0.65	*0.54*	1.83	0.46	1.19
**9**	5.19	0.65	*0.54*	1.83	0.46	1.19
**10**	5.83	0.67	*0.56*	1.87	0.44	1.19
**11**	6.45	0.63	*0.52*	1.79	0.48	1.19

[a] Decay time, [b] Fractional contribution, [c] Weighted average decay time $\tau_{av} = \Sigma (\tau_i \times \alpha_i)$.

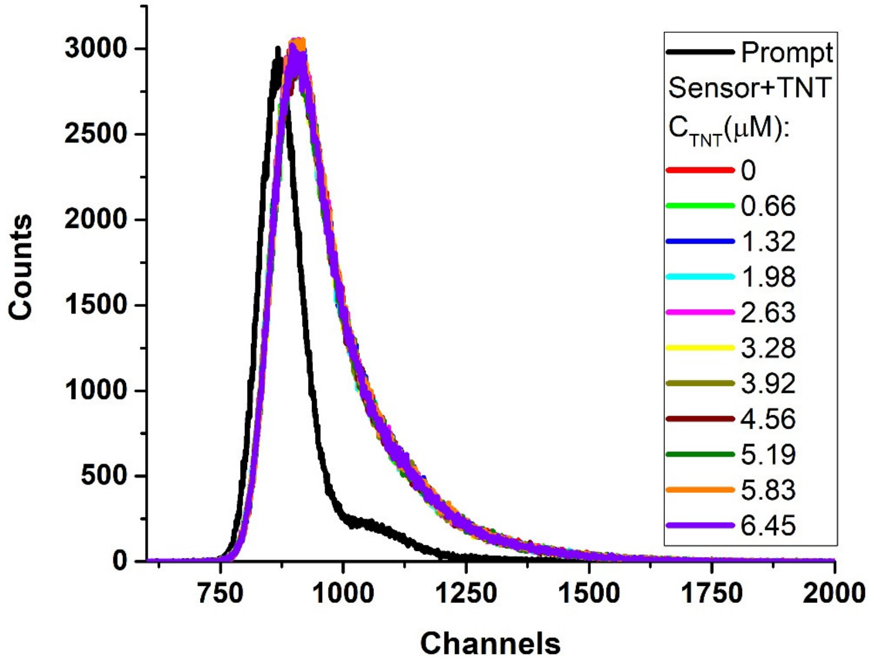

Figure S28. Overlayed time-resolved emission spectra for compound **3b** in the presence of TNT. Sample preparation: C(3b) = 1×10^{-6} M.

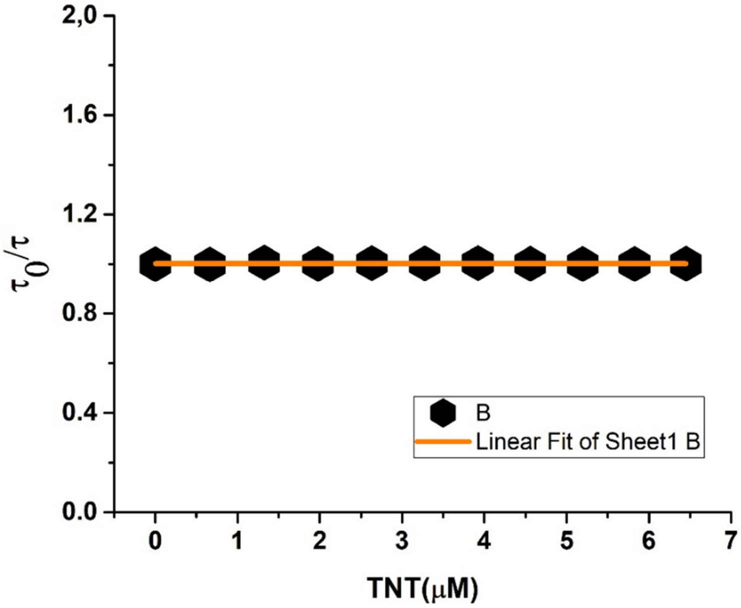

Figure S29. The graphical result of time-resolved fluorescence titration of **3b** by TNT monitored at 350 nm.

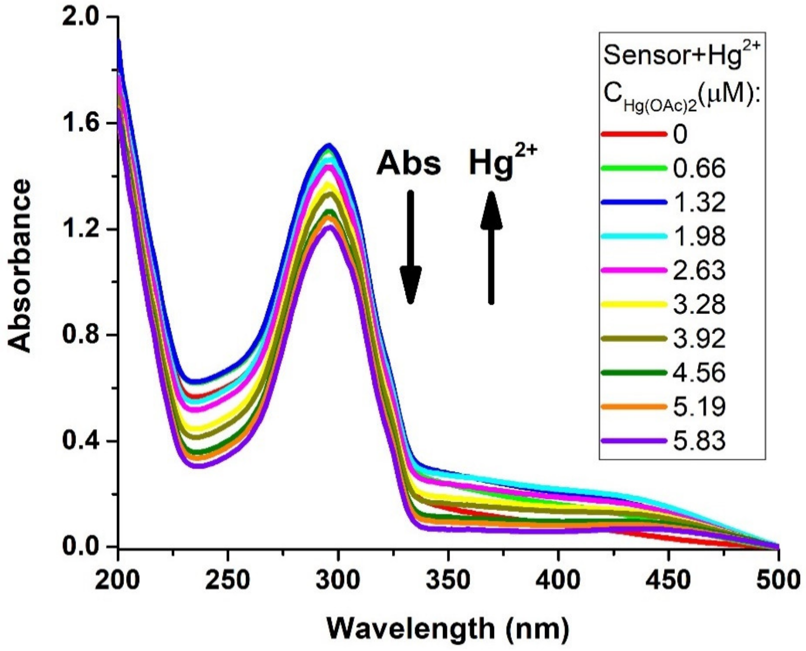

Figure S30. Overlayed absorption spectra for compound **3a** in the presence of Hg^{2+}. Sample preparation: C(3a) = 1×10^{-6} M.

Table S4. Lifetime measurement of probe **3a** in the presence of Hg^{2+} in MeCN:H_2O [90:10 (vol.%)] at r.t. (C(3a) = 1×10^{-6} M).

Entry	$C_{Hg(OAc)2}$, μM	τ_1, ns[a]	α_1[b]	τ_2, ns[a]	α_2[b]	τ_{av}, ns[c]
**1**	0	0.60	*0.72*	2.36	0.28	1.09
**2**	0.66	0.61	*0.72*	2.39	0.28	1.11
**3**	1.32	0.63	*0.72*	2.43	0.28	1.13
**4**	1.98	0.64	*0.73*	2.51	0.27	1.14
**5**	2.63	0.62	*0.72*	2.44	0.29	1.14
**6**	3.28	0.63	*0.71*	2.44	0.29	1.15
**7**	3.92	0.63	*0.70*	2.44	0.29	1.16
**8**	4.56	0.63	*0.70*	2.43	0.30	1.17
**9**	5.19	0.64	*0.70*	2.42	0.30	1.17
**10**	5.83	0.65	*0.70*	2.43	0.30	1.19
**11**	6.45	0.66	*0.70*	2.48	0.30	1.20

[a] Decay time, [b] Fractional contribution, [c] Weighted average decay time $\tau_{av} = \Sigma (\tau_i \times \alpha_i)$.

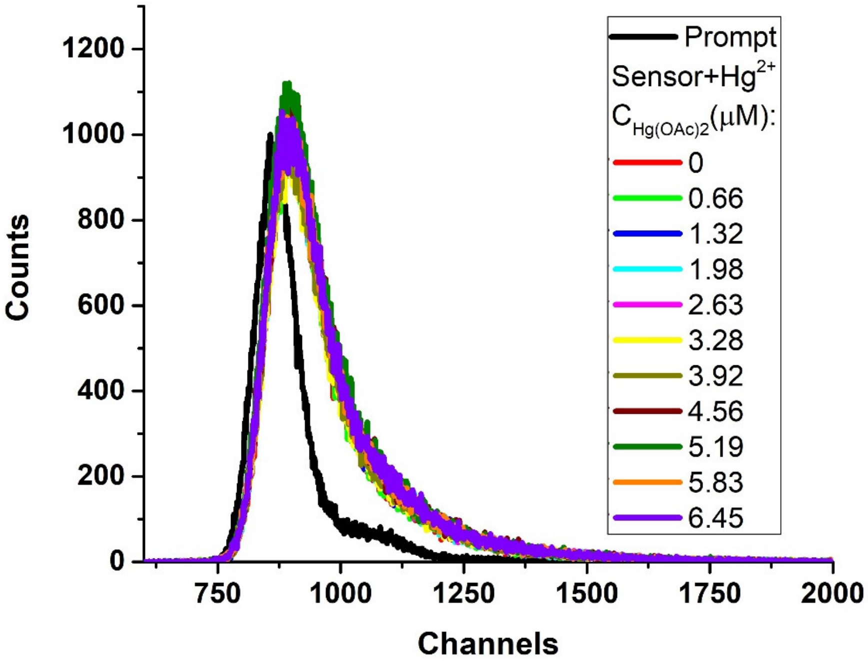

Figure S31. Overlayed time-resolved emission spectra for compound **3a** in the presence of Hg^{2+}. Sample preparation: C(3a) = $1{\times}10^{-6}$ M.

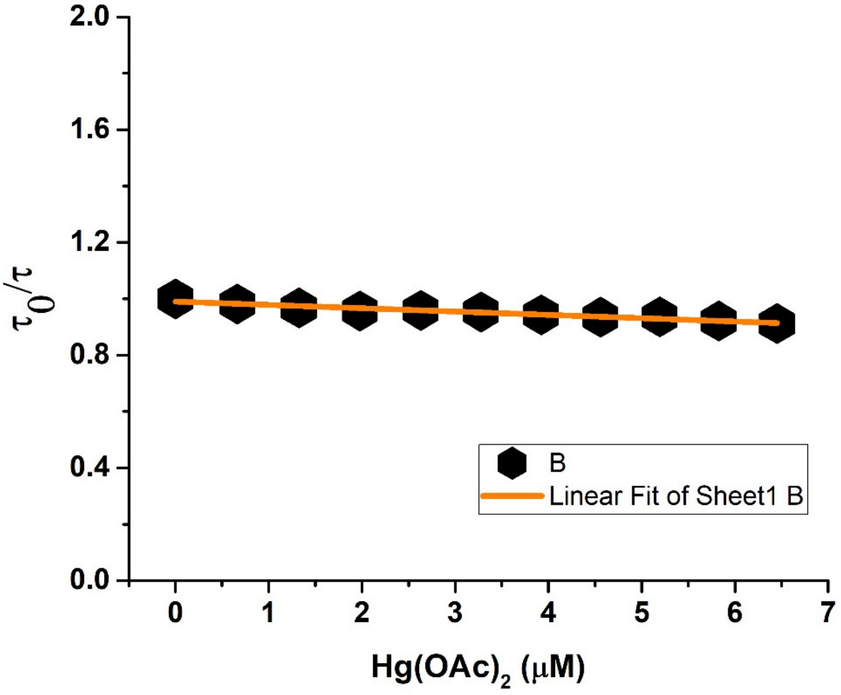

Figure S32. The graphical result of time-resolved fluorescence titration of **3a** by Hg^{2+} monitored at 350 nm.

7. DFT Calculations

The quantum chemical calculations were carried out at the CAM-B3LYP/6-31G*//PM6 and B3LYP/631pGs level of theory with the help of the Gaussian-09 [1] program package. No symmetry restrictions were applied during the geometry optimization procedure. The solvent effects were taken into account using the SMD (Solvation Model based on Density) continuum solvation model suggested by Truhlar and coworkers [2]. The Hessian matrices were calculated for all optimized model structures to prove the location of correct minima on the potential energy surface (no imaginary frequencies were

found in all cases). The Cartesian atomic coordinates for all optimized equilibrium model structures are presented in the attached xyz-files. The hole-electron analysis was carried out in Multiwfn program (version 3.7) [3].

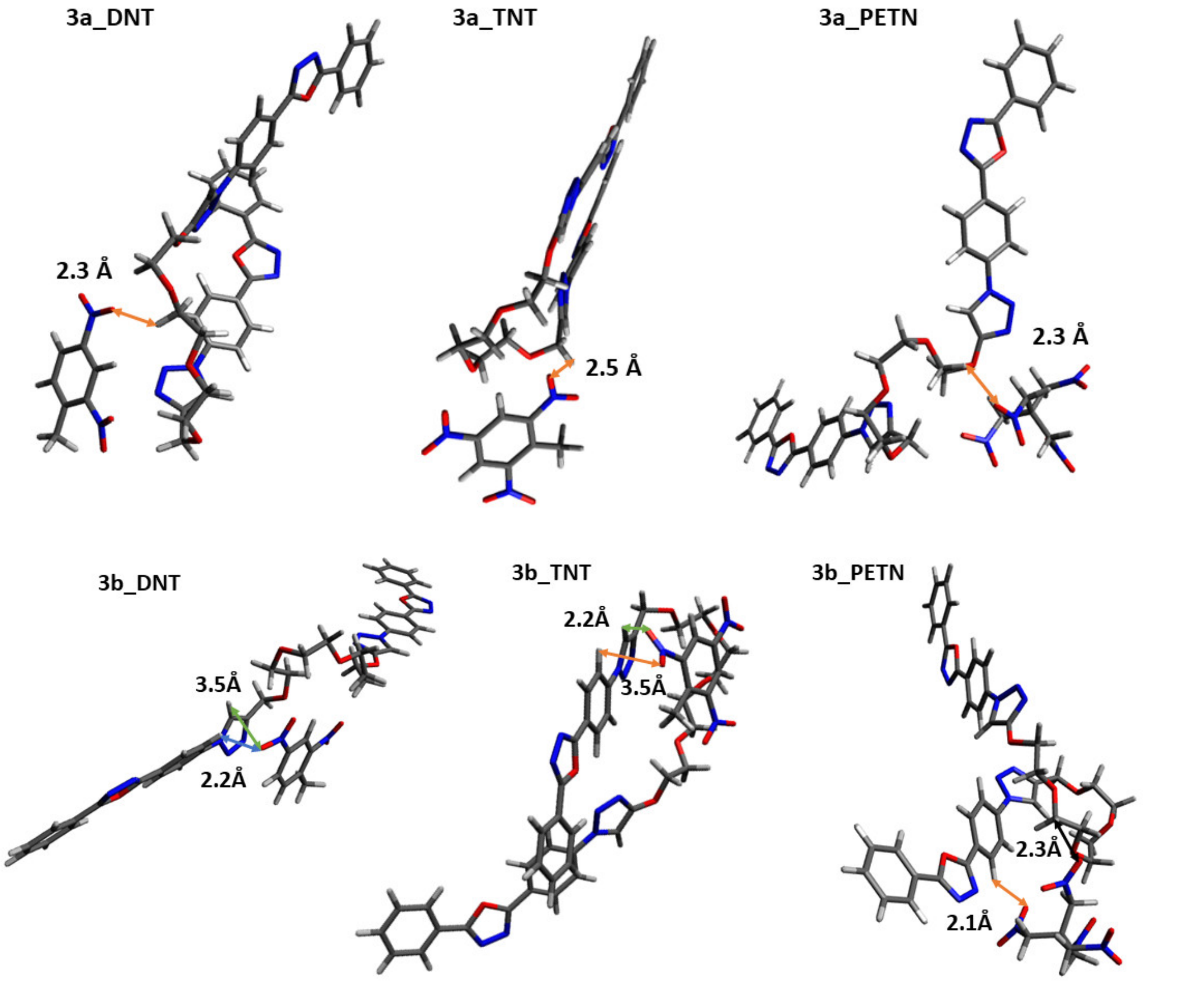

Figure S33. Geometric structures of the possible configurations for probes **3a,b** combining with molecular of analyte DNT/TNT/PETN (adducts): **3a_DNT, 3a_TNT, 3a_PETN, 3b_DNT, 3b_TNT, 3b_PETN** on ground state.

Table S5. Imaging of HOMO/LUMO of **3a,b** and analytes based on B3LYP/631pGs functional in gas phase.

**Compound**	**HOMO**	**LUMO**
**3a**	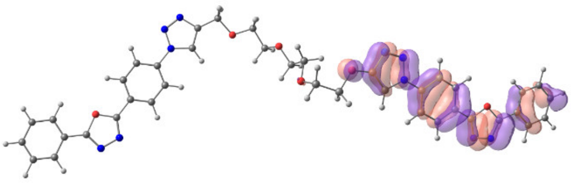	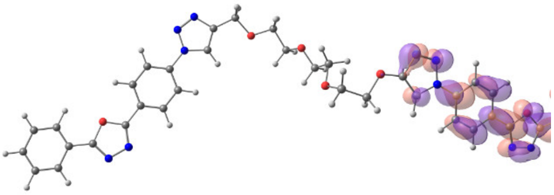
**3b**	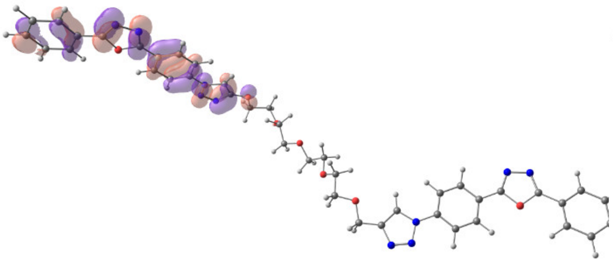	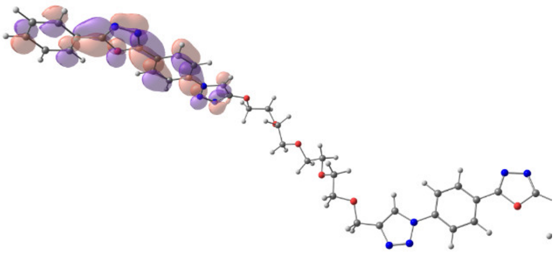
**DNT**	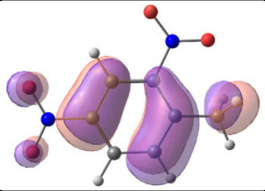	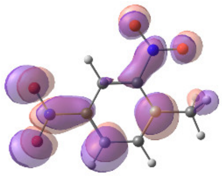
**TNT**	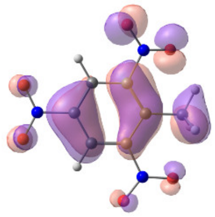	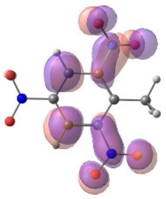
PETN	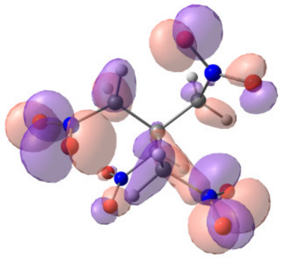	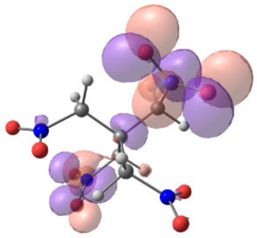

Table S6. The oscillator strengths for $S_0 \rightarrow S_1$ and $S_0 \rightarrow S_2$ transitions.

**Model structure**	**The oscillator strengths for $S_0 \rightarrow S_1$ and $S_0 \rightarrow S_2$ transitions**
**3a**	$S_0 \rightarrow S_1$ 1.6187 $S_0 \rightarrow S_2$ 1.4353
**3b**	$S_0 \rightarrow S_1$ 1.5808 $S_0 \rightarrow S_2$ 1.4674

8. Recognition of Hg^{2+} via Fluorescence "turn off" Process

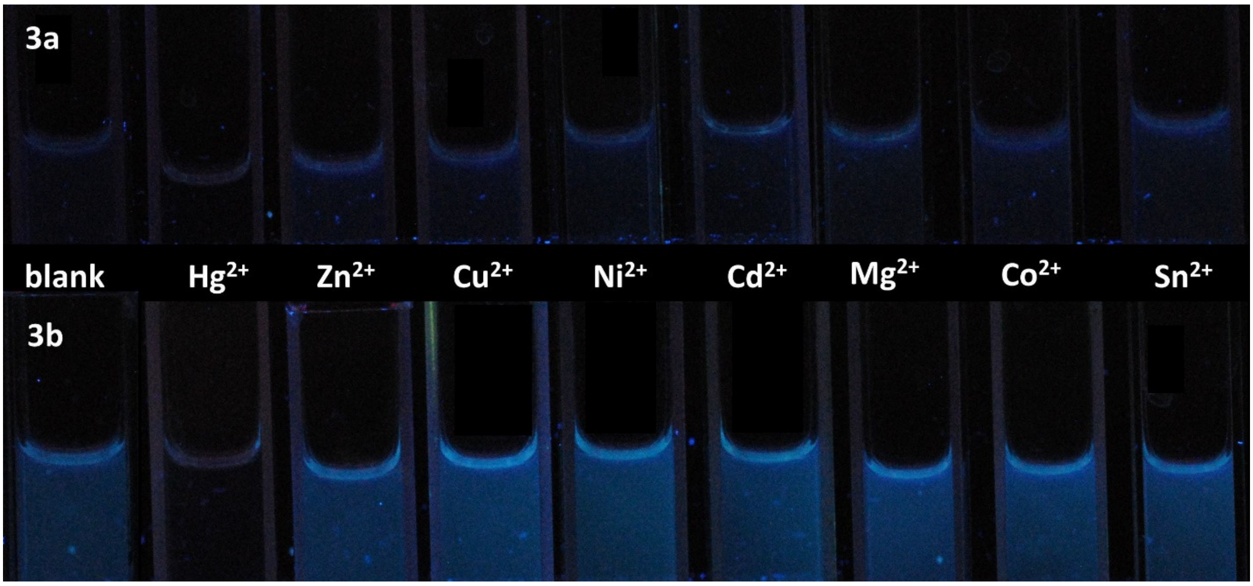

Figure S34. Qualitative assessment of the presence of metal ions by adding to the MeCN:H_2O [90:10 (vol.%)] solution of probes **3a** (upper row) and **3b** (bottom row) (2×10^{-4}M) aqueous solution of salt (2×10^{-2}M, ~1.0 eq.) under 365 nm UV light.

References

1. D.J.F. M. J. Frisch, G. W. Trucks, H. B. Schlegel, G. E. Scuseria, M. A. Robb, J. R. Cheeseman, G. Scalmani, V. Barone, B. Mennucci, G. A. Petersson, H. Nakatsuji, M. Caricato, X. Li, H. P. Hratchian, A. F. Izmaylov, J. Bloino, G. Zheng, J. L. Sonnenberg, M. Had, Gaussian 09, Revision C.01, Wallingford, CT. (2010).
2. A. V. Marenich, C.J. Cramer, D.G. Truhlar, Universal solvation model based on solute electron density and on a continuum model of the solvent defined by the bulk dielectric constant and atomic surface tensions, J. Phys. Chem. B. 113 (2009) 6378–6396. doi:10.1021/jp810292n**.**
3. Lu, T.; Chen, F. Multiwfn: A Multifunctional Wavefunction Analyzer. J. Comput. Chem. 2012, 33, 580–592, doi:10.1002/jcc.22885.
